# Effect of Aromatic System Expansion on Crystal Structures of 1,2,5-Thia- and 1,2,5-Selenadiazoles and Their Quaternary Salts: Synthesis, Structure, and Spectroscopic Properties

**DOI:** 10.3390/ma13214908

**Published:** 2020-10-31

**Authors:** Jan Alfuth, Beata Zadykowicz, Artur Sikorski, Tadeusz Połoński, Katarzyna Eichstaedt, Teresa Olszewska

**Affiliations:** 1 Department of Organic Chemistry, Gdańsk University of Technology, 80-233 Gdańsk, Poland; jan.alfuth@pg.edu.pl (J.A.); tadeusz.polonski@pg.edu.pl (T.P.); kat.eichstaedt@gmail.com (K.E.); 2 Luminescence Research Group, Faculty of Chemistry, University of Gdańsk, 80-308 Gdańsk, Poland; beata.zadykowicz@ug.edu.pl; 3 Laboratory of Crystallochemistry, Faculty of Chemistry, University of Gdańsk, 80-308 Gdańsk, Poland; artur.sikorski@ug.edu.pl

**Keywords:** chalcogenadiazoles, chalcogen bonds, crystal engineering, spectral properties

## Abstract

Rational manipulation of secondary bonding interactions is a crucial factor in the construction of new chalcogenadiazole-based materials. This article reports detailed experimental studies on phenanthro[9,10-*c*][1,2,5]chalcogenadiazolium and 2,1,3-benzochalcogenadiazolium salts and their precursors. The compounds were synthesized, characterized employing NMR and UV-Vis spectroscopy. TD-DFT calculations were also performed. The influence of the size of the aromatic system on the molecular motifs formed by the compounds in the solid state has been studied by means of single-crystal X-ray diffraction. In case of the salts, the nature of an anion was also taken into consideration. The results show that cyclic [E···N]_2_ supramolecular synthon connects neighboring molecules of phenanthro[9,10-*c*][1,2,5]chalcogenadiazoles, with a relatively large aromatic system, in dimers regardless of the chalcogen atom in the molecule. Both *N*-methyl-2,1,3-benzothiadiazolium and *N*-methylphenanthro[9,10-*c*][1,2,5]chalcogenadiazolium cations have a strong affinity for triflate and iodide anions, therefore the formation of S···N or Se···N secondary bonding interactions is observed only in two out of the eight quaternary salts. Less coordinating anions must be used to enable the building blocks studied to form cyclic [E···N]_2_ synthons. Moreover, for two of the triflate salts, which are isostructural, a new supramolecular motif has been observed.

## 1. Introduction

Due to their unique physical and chemical characteristics, molecules containing the 2,1,3-benzothiadiazole and/or 2,1,3-benzoselenadiazole unit are mainly known as components used in construction of optoelectronic devices, including organic light-emitting diodes (OLEDs), solar cells, and liquid crystals [1,2,3,4,5,6,7]. Apart from that, these systems are also building blocks in crystal engineering [8], self-assembly of supramolecular capsules [9,10], macrocycles, and polymers [11].

The presence of two nitrogen atoms separated by a heavy p-block element (sulfur or selenium) in their structures enables these molecules to aggregate through chalcogen bonding [12,13,14,15], which is a subgroup of non-covalent interactions which has received growing attention in the last two decades. Chalcogen bonds (ChBs) result from interaction between an electrophilic region of a Group VI element (i.e., S, Se, Te) and a Lewis base. Due to their strength and directionality (comparable to hydrogen and halogen bonds) ChBs are intriguing alternatives to the predominant hydrogen bonding in various areas of research including crystal engineering, organic reactivity and catalysis [16,17], as well as anion transport and recognition [13,18,19].

**Figure 1:**
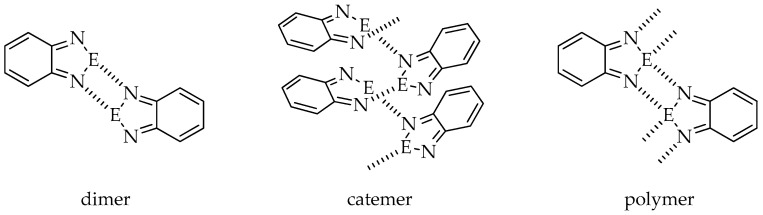
Possible ways of molecule aggregation in crystals of 2,1,3-benzochalcogenadiazoles (E = S, Se, Te).

In the absence of other Lewis bases, molecules of 2,1,3-benzochalcogenadiazoles and their derivatives aggregate via E···N interactions (E = S, Se, Te). Depending on the substituent attached to the benzene ring, the heterocycles of one molecule can contact with a neighboring heterocyclic ring building dimers, catemers or polymers (Figure 1) through the formation of E···N interactions. Dimers and polymers are stabilized by cyclic four-membered supramolecular synthons [E···N]_2_.

Recently, it has been proved that blocking one of the nitrogen atoms in a molecule of 2,1,3-benzochalcogenadiazole, either by attachment of a transition metal ion (Hg [20], Cu, Ag, Co [21]) or *N*-alkylation, shortens the E···N intermolecular distance and consequently strengthens chalcogen bonding [22]. Thus, monoalkylated 2,1,3-benzochalcogenadiazolium cations are very attractive supramolecular building blocks for the design of ChB systems. They can potentially replace 2,1,3-benzotelluradiazole derivatives in supramolecular architecture which are known to create strong intermolecular [Te···N]_2_ synthons in both solids and solutions [23,24,25]. In comparison to sulfur and selenium, tellurium forms stronger ChBs. However, telluradiazoles are usually susceptible to oxidative decomposition, thus restricting the incorporation of such elements into more complex systems. Moreover, the simple synthesis of monoalkylated 2,1,3-benzochalcogenadiazolium salts and their stability towards hydrolysis and oxidation is an additional advantage.

**Figure 2:**
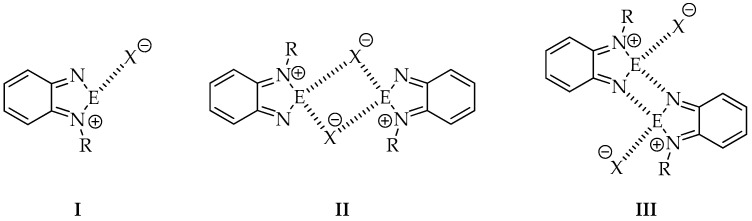
Most common motifs observed in crystals of *N*-alkyl-2,1,3-benzochalcogenadiazolium salts.

In comparison to the parent neutral 2,1,3-benzochalcogenadiazoles, self-assembly of their *N*-alkylated derivatives is limited only to dimer formation in the best case. It is due to the presence of a substituent, which blocks one side of the molecule. It is worth noticing that two main factors, i.e., the size and nature of the alkyl group and the anion play a crucial role in self-association of *N*-alkylated cationic rings in solid. So far a few motifs recurring in crystals of chalcogenadiazolium salts have been identified [26] (Figure 2).

For example, pattern number **I** has been observed in *N*-methyl-2,1,3-benzothiadiazolium triflate and pattern **III** in *N*-methyl-2,1,3-benzoselenadiazolium triflate described by Risto and co-workers [22]. Vargas-Baca et al. [26], who studied both theoretically and experimentally a series of *N*-alkyl-2,1,3-benzoselenadiazolium iodides with methyl, isopropyl and *tert*-butyl substituents, also identified the above mentioned motifs.

It has to be emphasized that despite knowing the motifs recurring in crystals of chalcogenadiazolium salts we are still not able to predict the combination of ions (cation and anion) which leads to their specific arrangements in the solid state. One way to accomplish this is through a systematic investigation series of quaternary salts of chalcogenadiazoles with the same cationic ring and a different counterion or the other way round.

**Figure 3:**
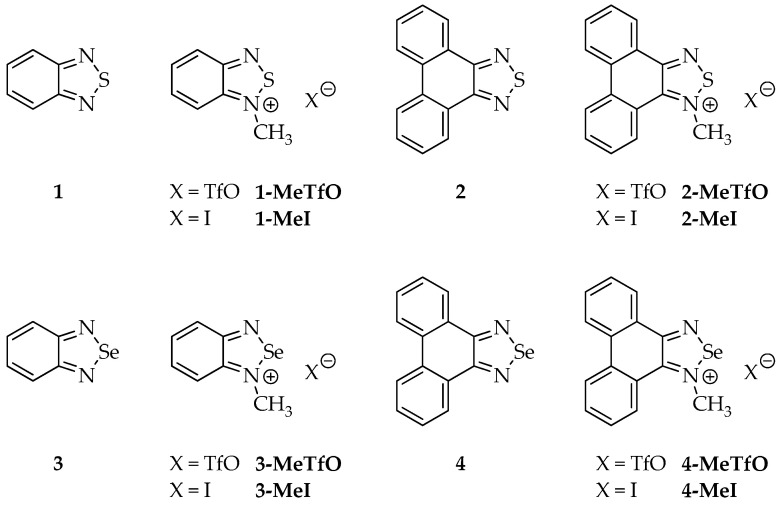
Structures of all studied compounds.

Therefore, we have synthesized a series of triflate and iodide salts of *N*-methylated 2,1,3-benzochalcogenadiazoles and *N*-methylated phenanthro[9,10-*c*][1,2,5]chalcogenadiazoles (Figure 3) and we examined their crystal structures. To limit competing forces in the crystal lattice the selected cationic rings were devoid of any functional groups and differed only in the size of the *π*-aromatic system.

Here we discuss the influence of both enlarging the aromatic system in cations and anion exchange on the molecular motifs formed in the quaternary salts. Spectroscopic properties (UV-Vis) useful for the characterization of these species in solution and solid state along with time-dependent density functional theory (TD-DFT) calculations are also reported.

## 2. Materials and Methods

### 2.1. Synthesis

The structures of all the studied compounds are shown in Figure 3.

**Figure 4:**
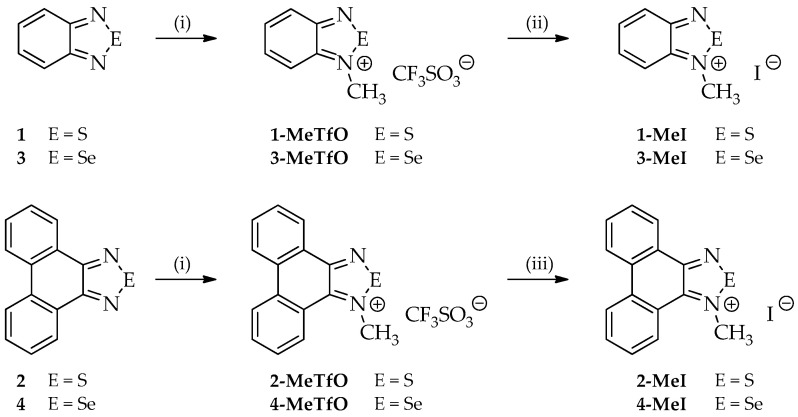
Syntheses of all compounds: (**i**) CF_3_SO_3_CH_3_, dry (CH_2_Cl)_2_, 60 °C; (**ii**) Bu_4_NBr, MeOH/toluene; and (**iii**) NaI, acetone.

Four out of the twelve studied compounds (Figure 3), namely **2-MeTfO**, **2-MeI**, **4-MeTfO** and **4-MeI**, are new and have been synthesized for the first time. 2,1,3-Benzothiadiazole (**1**), phenanthro[9,10-*c*][1,2,5]thiadiazole (**2**), 2,1,3-benzoselenadiazole (**3**) and phenanthro[9,10-*c*][1,2,5]selenadiazole (**4**) were prepared following the reported procedures [27,28,29]. *N*-Methyl-2,1,3-benzochalcogenadiazolium triflates (**1-MeTfO** and **3-MeTfO**) were also synthesized according to the reported procedure [22]. The remaining triflates (**2-MeTfO** and **4-MeTfO**) were obtained analogously (Figure 4). Iodides were prepared by reaction of a triflate with an excess of tetrabutylammonium iodide in MeOH/toluene (**1-MeI** and **3-MeI**) or NaI in acetone (**2-MeI** and **4-MeI**). For experimental details and NMR spectra see Supplementary Materials (Experimental Procedures, pages S1–S2 and Figures S1−S20, respectively).

*N*-Methyl-2,1,3-benzothiadiazolium iodide (**1-MeI**) and selenadiazole **4** have been synthesized earlier [28,29], however their crystal structures had not been determined. The structures of the other compounds (**1**–**3**, **1-MeTfO**, **3-MeTfO**, **3-MeI**) have been already reported. We have synthetized them and determined their crystal structures once more to have the whole set of structures for comparison.

### 2.2. X-ray Measurements and Refinements

Good-quality single-crystal specimens of **1-MeI**, **2-MeTfO**, **2-MeI**, **4**, **4-MeTfO** and **4-MeI** were selected for the X-ray diffraction experiments at T = 295(2) K. They were mounted with epoxy glue at the tip of glass capillaries. Diffraction data were collected on an Oxford Diffraction Gemini R ULTRA Ruby CCD diffractometer (Oxford, England) with MoKα (λ = 0.71073 Å) radiation. In all cases, the lattice parameters were obtained by least-squares fit to the optimized setting angles of the reflections collected by means of CrysAlis CCD (Version 1.171.36.28) [30]. Data were reduced using CrysAlis RED software (Version 1.171.36.24) [30] and applying multi-scan absorption corrections (empirical absorption correction using spherical harmonics, implemented in the SCALE3 ABSPACK scaling algorithm). The structural resolution procedure was carried out using the SHELX package [31]. The structures were solved with direct methods that carried out refinements by full-matrix least-squares on *F*^2^ using the SHELXL-2017/1 program [31]. All H-atoms bound to aromatic C-atoms were placed geometrically and refined using a riding model with C–H = 0.93 Å and U_iso_(H) = 1.2U_eq_(C). All H-atoms from the methyl group were positioned geometrically and refined using a riding model, with C–H = 0.96 Å and U_iso_(H) = 1.5U_eq_(C). All interactions were calculated using the PLATON program (Version 181115) [32]. The ORTEP II [33], PLUTO-78 [34] and Mercury (Version 4.2.0) [35] programs were used to prepare the molecular graphics.

### 2.3. DFT Calculations

The molecular structures of the neutral form of **1**–**4** and their methylated cationic forms (**[1-Me]^+^**, **[2-Me]^+^**, **[3-Me]^+^**, **[4-Me]^+^**) were optimized at the DFT [36] level of theory with the B3LYP-D3 functional [37,38] and the 6-31++G(d,p) basis set [39,40]. After completion of each optimization, the Hessian (second derivatives of the energy as a function of nuclear coordinates) was calculated and checked for positive definiteness to assess whether the structures were true minima. Next, for geometries obtained at the DFT level, the UV-Vis spectra were calculated using the TD-DFT [41] method with the same functional and basis set. For all investigated molecules, 150 states were computed. The solvent effect was included with the full geometry optimizations utilizing the Polarized Continuum Model (PCM) (UAHF radii were used to obtain the molecular cavity) [42,43]. All calculations were performed using the Gaussian16 suite (Revision C.01) [44], whereas the ChemCraft program (Version 1.8) [45] was utilized to visualize the equilibrium structures of the molecules.

## 3. Results and Discussion

### 3.1. X-ray Crystallography

Single-crystal X-ray diffraction measurements show that 2,1,3-benzochalcogenadiazoles **1** and **3** crystallize in orthorhombic *Pna*2_1_ space group with one molecule in the asymmetric unit and are isostructural [46,47]. Unsurprisingly, **1** does not form the [E···N]_2_ synthons. Its molecules are linked via single S···N chalcogen bonds forming catemeric structures (resembling helices) along the *c*-axis stabilized by *π–π* interactions (Supplementary Materials Figure S23a). What is surprising is that **3** does not form these motifs either, but its molecules aggregate in the same way as in **1** (Supplementary Materials Figure S23b). Interatomic distances between atoms engaged in the chalcogen bonds are: *d*_S···N_ = 3.22 Å and *d*_Se···N_ = 3.16 Å for compounds **1** and **3**, respectively, and are shorter than the sum of the van der Waals radii of sulfur and nitrogen atoms (3.35 Å) and selenium and nitrogen atoms (3.45 Å) (*δ*_%_ = 96% and 92%, respectively) (Table 1).

**Figure 5:**
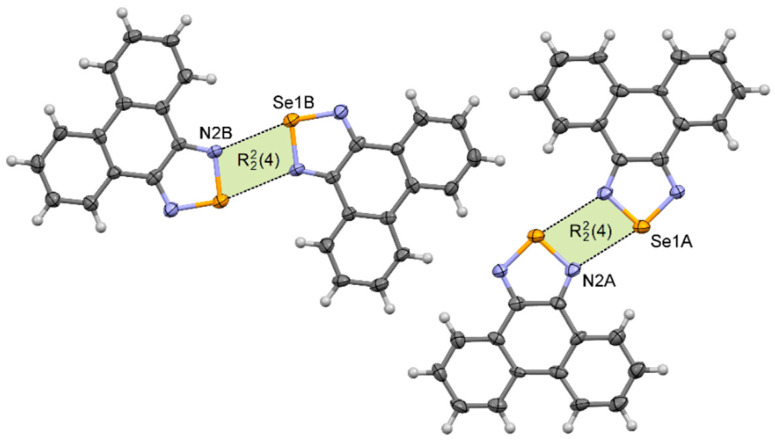
Arrangement of molecules in the crystal of **4**.

Phenanthro[9,10-*c*][1,2,5]thiadiazole **2** [48] crystallizes in the monoclinic *P*2_1_/*n* space group with one molecule in the asymmetric unit, whereas phenanthro[9,10-*c*][1,2,5]selenadiazole **4** crystallizes in the triclinic *P* –1 space group with two molecules in the asymmetric unit. Despite different crystallographic parameters, their crystal structures are very similar (Supplementary Materials Figures S24 and S25). In their crystals the neighboring molecules are linked via two E···N chalcogen bonds forming the [E···N]_2_ synthons. In each pair (dimer) molecules are very nearly coplanar. The length of the chalcogen bond in **2** is *d*_S···N_ = 3.22 Å, which is 8% shorter than the van der Waals radii of both atoms (Table 1). In the crystal of **4**, two different [E···N]_2_ synthons produced by each molecule in the asymmetric unit were observed (Figure 5). For molecules *A* the Se···N distance is 2.91 Å, whereas for molecules *B* it is 2.97 Å (*δ*_%_ = 84% and 86%, respectively). The neighboring dimers **2**_2_ and **4**_2_ interact through *π–π* stacking interactions to produce blocks along the *a*-axis.

Reaction of **1**–**4** with methyl triflate yields their quaternary salts. The reaction causes a desymmetrization of a parent molecule and often leads to the breakage of the [E···N]_2_ synthon in favor of the formation of new ones.

**Figure 6:**
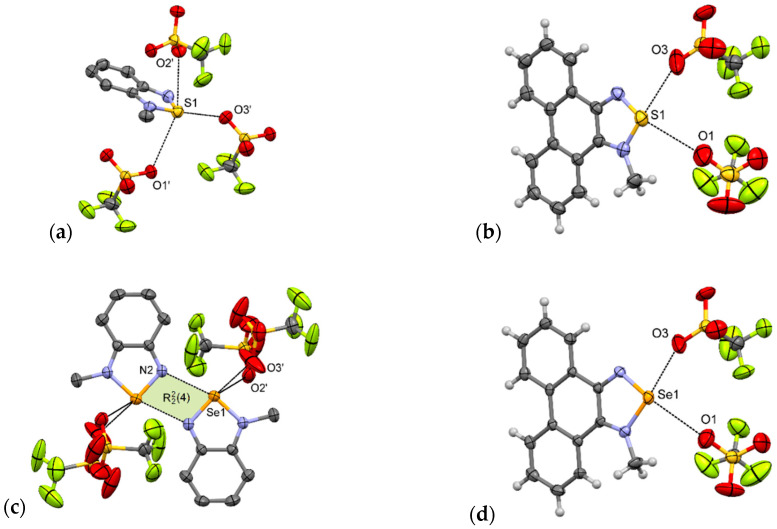
Arrangement of ions in the crystals of: (**a**) **1-MeTfO**, (**b**) **2-MeTfO**, (**c**) **3-MeTfO**, and (**d**) **4-MeTfO** (some hydrogen atoms are omitted for clarity).

Indeed, in **1-MeTfO** [22] there are no [E···N]_2_ synthons and the sulfur atom of the *N*-methyl-2,1,3-benzothiadiazolium cation interacts with three trifluoromethanesulfonate anions via S···O interactions. The shortest interaction (*d*_S1···O3_ = 2.82 Å, *δ*_%_ = 85%) is opposite the quaternary nitrogen in a motif resembling **I** (Figure 6a). The other two contacts are 3.23 and 3.28 Å and lie almost perpendicular to the aromatic system. As a consequence of these interactions, in the crystal packing of **1-MeTfO** there are corrugated layers of connected cations and anions along the *a*-axis. The layers are linked through *π–π* interactions between aromatic rings.

**3-MeTfO** [22] is not isostructural with **1-MeTfO**. In the crystal of **3-MeTfO**, the [E···N]_2_ synthons are preserved and *N*-methyl-2,1,3-benzoselenadiazolium cations are linked by Se···N interactions (Figure 6c). The arrangement of ions resembles motif **III**. The thing that distinguishes it from the motif is that the selenium atom interacts with two oxygen atoms from two different anions. The Se···O bonds are of different lengths (Table 2). The shortest interaction is predictably Se···N ChB (*d* = 2.71 Å, *δ*_%_ = 79%). As in **1-MeTfO**, the crystal of **3-MeTfO** consists of layers of connected cations and anions along the *a* axis. The neighboring layers are linked through *π–π* interactions between aromatic rings of cations.

Compounds **2-MeTfO** and **4-MeTfO** are isostructural. The type and number of interactions are practically identical (Figure 6b,d). There are two E···O chalcogen bonds varying in strength: the stronger one opposite to a quaternary nitrogen and associated with *σ*-hole [49,50] with a greater electrostatic potential, and a much weaker one opposite to the other nitrogen atom. To the best of our knowledge it is a new supramolecular motif never observed before for this class of compounds. The bond parameters are listed in Table 2. Predictably, Se···O interactions are slightly shorter than S···O. Contact between atoms Se1 and O3 (*d* = 2.65 Å, *δ*_%_ = 77%) is the shortest ChB of all those discussed in this paper. The neighboring chalcogenadiazolium cations interact through *π–π* stacking interactions producing blocks along the *a*-axis (Supplementary Materials Figures S26 and S27).

Taking into account the fact that for quaternary salts the shape and size of an anion largely determine their crystal structure, the dumbbell-shaped trifluoromethanesulfonate anion was replaced with a ball-shaped halide ion. Theoretically, such symmetry of the counterion allows for the formation of more supramolecular synthons. In the case of the following salts, an anion exchange leads precisely to this effect.

**Figure 7:**
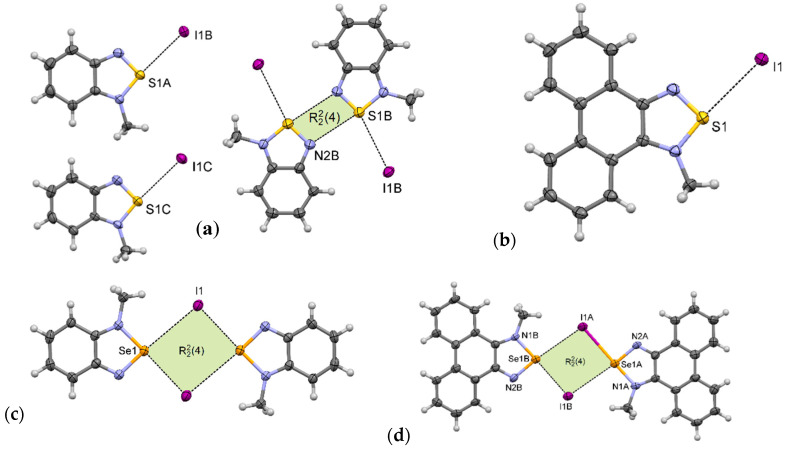
Arrangement of ions in the crystals of: (**a**) **1-MeI**, (**b**) **2-MeI**, (**c**) **3-MeI**, and (**d**) **4-MeI** (some hydrogen atoms are omitted for clarity).

**1-MeI** crystallizes in the monoclinic *P*2_1_/*c* space group with three cations and three anions in the asymmetric unit. In its crystal structure there are pairs of associated benzothiadiazolium cations (as in motif **III**); however, the whole structure is more complicated. Only one of the three cations forms the [S···N]_2_ synthon. The other two are arranged in motif **I** (Figure 7a). In the crystal packing, weak C–H···I and C–H···N hydrogen bonds and *π–π* interactions are also present (Figure S28).

The crystal structure of **2-MeI** is much simpler than that of **1-MeI**. **2-MeI** crystallizes in the monoclinic *P*2_1_/*n* space group with one cation and one anion in the asymmetric unit. In its crystals the ions are linked via S···I interactions forming supramolecular motif **I** (Figure 7b). These contacts together with weak C–H···I hydrogen bonds create overlapping undulating polymeric chains along the *b*-axis (Figure S29). The neighboring chains are linked through *π–π* stacking interactions between aromatic rings.

Unlike the thiadiazolium iodides, **3-MeI** [26] and **4-MeI** form [Se···I]_2_ synthons (motif **II**). **3-MeI** crystallizes with one cation and one anion in the asymmetric unit. The ions form centrosymmetric [Se···I]_2_ synthon with distances between the Se atom and iodide anion equal to 3.18 Å (*δ*_%_ = 82%) and 3.61 Å (*δ*_%_ = 93%) (Figure 7c). Unexpectedly, in the crystal of **4-MeI** the synthon is non-centrosymmetric with four different Se···I distances (Figure 7d), the shortest being 3.07 Å (*δ*_%_ = 79%), which is exceptionally low value for this type of interaction (Table 3). Crystal packing of **4-MeI** is presented in Figure S30.

Selected crystallographic data of **1-MeI**, **2-MeTfO**, **2-MeI**, **4**, **4-MeTfO**, and **4-MeI** are listed in Table S1 in the Supplementary Material.

### 3.2. UV-Vis Spectra

**Figure 8:**
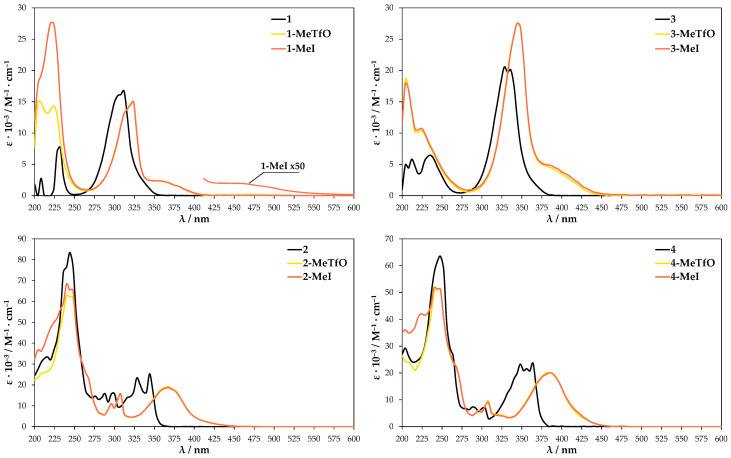
UV-Vis spectra of the compounds dissolved in methanol.

UV-Vis absorption spectra were further conducted to provide more information on the self-assembly process. They were conducted in both the solid state and solution. The neutral molecules **1**–**4** in methanol solution at room temperature show two major absorption bands in the range of 230–250 nm and 310–360 nm (Figure 8). The maxima bands for **3** and **4** are shifted about 25 nm to the red in comparison to **1** and **2** due to the presence of heavier chalcogen (selenium atom) in their structures. These spectra are in reasonable agreement with TD-DFT calculation for **1**–**4**, which attributes the second maximum absorption band to HOMO → LUMO transition (Supplementary Materials, Figure S21). The tabulated results of calculated UV-Vis spectra and oscillator strength values are given in Supplementary Materials Table S2. In comparison to the parent molecules **1**–**4** the absorption spectra of methanolic quaternary salt solutions display obvious changes since the aromatic cations have an asymmetric structure. The methyl substituent influences the electron cloud distribution of LUMO and the overlapping of the frontier molecular orbitals (FMOs). Compounds **1-MeTfO**, **1-MeI**, **3-MeTfO**, and **3-MeI** show two relatively strong bands and a weak one, whose maxima are at about 225, 325, and 375 nm for *N*-methyl-2,1,3-benzothiadiazolium salts and 225, 350, and 400 nm for *N*-methyl-2,1,3-benzoselenadiazolium salts. Moreover, it is worth noticing that the shape of two absorption peaks at the longer wavelength in pairs **1-MeTfO**, **1-MeI** and **3-MeTfO**, **3-MeI** are identical. This is due to the fact that the spectra were recorded in diluted solution of the salts in which cation-anion interactions are relatively weak and their influence can be neglected. Thus, the spectra reflect only the cationic structure, which is the same.

The UV-Vis spectra of **2-MeTfO**, **2-MeI**, **4-MeTfO**, and **4-MeI** salts show two major absorption bands in the range of 230–250 nm and 370–390 nm. The peaks at the longer wavelength are prominently red shifted in comparison to the neutral compounds **2** and **4**. The spectrum of **1-MeI** measured at high salt concentration display an additional weak and broad absorption band at 460 nm.

The TD-DFT calculation results for the four investigated cations in methanol are compiled in Table S3. They predict an absorption band in the range of 370–390 nm, which results from HOMO–1 → LUMO excitation, which is related to the electron density shift to nitrogen atoms and the weak one above 400 nm results from HOMO → LUMO transition (Supplementary Materials, Figure S22). Whereas the absorption between 370 and 390 nm is consistent with the experimental results, the weak band above 400 nm is not present in the measured spectra. The reason for it is probably the difference in the theoretical and experimental conditions. A single cation surrounded by a single layer of solvent is used in theoretical work. It is quite different from the experimental condition, in which the anion–cation interaction as well as the anion type do play a role.

**Figure 9:**
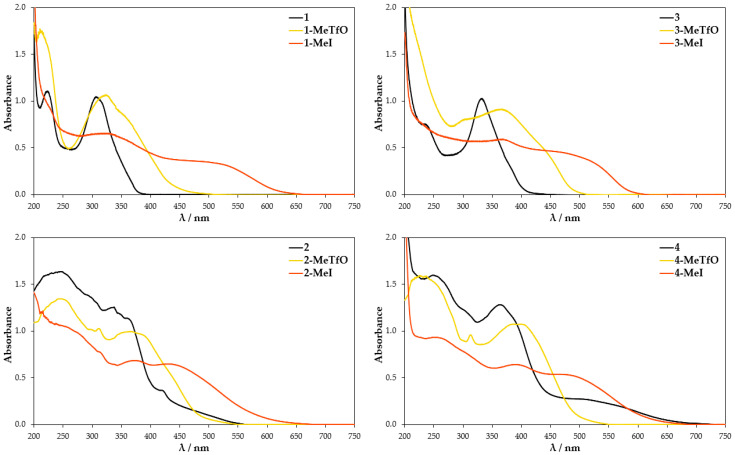
Absorption spectra of the compounds measured in KBr disks.

As anticipated, the UV-Vis spectra of the compounds measured in the solid (Figure 9) do not resemble their analogues taken in solutions. Compounds **1** and **3** show two major absorption bands in the region of 215–225 nm and 330–350 nm, whereas the molecules of **2** and **4** with an extended *π*-system display three bands: two strong ones (λ_max_ = 250 nm and 350 nm for **2** and λ_max_ = 250 nm 375 nm for (**4**) and one weak shoulder peak (at 475 nm for **2** and at 525 nm for (**4**). The main difference is seen in the spectra of quaternary salts due to the fact that they reflect the arrangement of molecules and their interactions in crystals. In the spectra of all iodide salts, namely **1-MeI**, **2-MeI**, **3-MeI**, and **4-MeI**, electron transitions at longer wavelength in the region of 490–525 nm are observed. By contrast absorption bands in that region appear neither in the spectra of the triflate salts nor neutral compounds **1**–**4**. On the other hand, an identical supramolecular [Se···I]_2_ synthon connects molecules in the crystals of **3-MeI** and **4-MeI.** Similarly, in the crystals of **1-MeI** and **3-MeI**, ions are linked via S···I interactions. Thus, the longer wavelength absorption band can unequivocally be assigned to the *π* ← I^−^ anion–cation charge transfer. It is worth mentioning that the studied compounds are of different color: **1**–**4** are white or slightly yellow, the triflate salts are deep yellow and the iodides are brick-red to deep red. The colors disappear upon dissolution of the crystals. The observation is consistent with the UV-Vis spectra of iodides, in which the *π* ← I^−^ anion–cation charge transfer is observed only in the solid state (as in the diluted solutions the transition is no longer possible).

In the course of the investigation the electronic changes of 2,1,3-benzochalcogenadiazoles and their quaternary salts upon ring enlargement were also estimated. These changes were investigated by calculating the global reactivity descriptors [51,52,53,54,55] such as HOMO–LUMO gaps, electronegativity (χ), electron affinity (EA), ionization potential energy (IP), dipole moment and chemical hardness (η), softness (S). Global reactivity descriptors were calculated using equations presented in the Supplementary Materials (TD-DFT Calculations, page S14). The results are presented in Table 4.

Global reactivity is considered as the chemical behavior of a substance and is useful to understand chemical bonding, kinetic stability of the molecule and reactive sites in a molecular structure [51]. One of the valuable parameters used to get an insight into molecular chemical reactivity is the HOMO–LUMO gap energy. The results show that this parameter is technically the same for all neutral molecules **1**–**4** and its value is between 4.02 and 4.22 eV. The difference is more apparent in the series of cations. HOMO–LUMO gap decreases with an increased size of the aromatic unit from 3.69 eV for **[1-Me]^+^** to 3.01 eV for **[2-Me]^+^** and from 3.54 eV for **[3-Me]^+^** to 2.94 eV for **[4-Me]^+^**. Moreover, taking into account heterocycles of the same size, its value is slightly lower for those containing selenium atom.

The energy gap value is inversely linked with two other parameters, namely softness and hardness of molecules. Thus, molecules having a small energy gap are taken as soft molecules prone to change in the electronic configurations and associated with higher reactivity and less kinetic stability. In contrast to this, molecules having a larger energy gap are considered hard molecules resistant to change in the electronic configurations characterized by higher kinetic stability and lower chemical reactivity. **[1-Me]^+^** has the highest value of hardness and the lowest value of softness of the compounds (Table 4). Thus, cation **[1-Me]^+^** is the most stable, hard and less reactive. On the other hand, the chemical hardness of **[4-Me]^+^** is the smallest of all the compounds. Therefore, it is more reactive than others.

The results show that ionization energy (IP) and electron affinity (EA), which are descriptors related to electronegativity and chemical reactivity, decrease with an increase in conjugate *π* system in neutral molecules **1**–**4** as well as cations **[1-Me]^+^**‒**[4-Me]^+^**. Thus, the species **1**, **3**, **[1-Me]^+^** and **[3-Me]^+^** are better electron acceptors than **2**, **4**, **[2-Me]^+^** and **[4-Me]^+^**, respectively.

## 4. Conclusions

A detailed analysis of the crystal structures of the compounds clearly indicates that enlarging the aromatic system in the neutral molecules of 2,1,3-benzochalcogenadiazoles by two benzene rings leads to the formation of dimers, which are connected by [E···N]_2_ supramolecular synthon regardless of the chalcogen atom (sulfur or selenium). *N*-Methylation of 2,1,3-benzochalcogenadiazoles and phenanthro[9,10-*c*][1,2,5]chalcogenadiazoles yields their quaternary salts, in which the main motif occurring in the solid strongly depends on the nature of the anion.

In general, less coordinating, asymmetrical and large by volume trifluoromethanesulfonate anions prevent dimer aggregation and promote the formation of polymeric layer structures. Only in one out of the four triflate salts, namely **1-MeTfO**, the cations are linked by Se···N interactions to produce [Se···N]_2_ synthons. Furthermore, for two salts, i.e., **2-MeTfO 4-MeTfO**, which are isostructural, new supramolecular motif was observed. It is formed by two trifluoromethanesulfonate anions and one *N*-methylphenanthro[9,10-*c*][1,2,5]chalcogenadiazolium cation, which associate by two S···O (**2-MeTfO**) or Se···O (**4-MeTfO**) chalcogen bonds varying in strength.

An iodide, isotropic, monatomic anion, interferes with the formation of [E···N]_2_ ChBs to a greater extent. As a result, in the supramolecular architectures of iodide salts this synthon basically is not observed. Moreover, the nature of the chalcogen atom present in the heterocyclic cation quite substantially influences aggregation morphology. Thus, the sulfur atom present in *N*-methyl-2,1,3-benzothiadiazolium and *N*-methylphenanthro[9,10-*c*][1,2,5]thiadiazolium cations (**1-MeI** and **2-MeI**) leads to S···I interactions between oppositely charged ions, whereas the presence of the selenium atom in **3-MeI** and **4-MeI** results in the formation of dimeric units connected through [Se···I]_2_ synthons.

Finally, absorption spectra of all the compounds were computed and the calculated parameters compared with the experimental ones. The results show that perturbations to the electronic structure of the symmetric chalcogenadiazoles **1**‒**4** by introducing the methyl substituent lead to shift absorption bands in resulting cations to longer wavelength. In addition, an absorption band near 500 nm, which was assigned to the *π* ← I^−^ anion–cation charge transfer in the UV-Vis spectra of iodides **1-MeI**‒**4-MeI** taken in the solid states could be used as a sensor for E···I interaction present in crystal structures formed by these building blocks.

## Figures and Tables

**Table 1:** Comparison of the sums of van der Waals radii, length (*d*), valence angles (θ) and parameters *δ*_%_ of ChBs occurring in the crystal structures of studied 1,2,5-chalcogenadiazoles (double values refer to two interactions differing in energy).

	1	2	3	4
$r_{{\mathrm{vdW}}_{E}}+r_{{\mathrm{vdW}}_{N}}$ (Å)	3.35	3.35	3.45	3.45
$d_{E\cdot \cdot \cdot N}$ (Å)	3.22	3.09	3.16	2.91; 2.97
$\theta_{N-E\cdot \cdot \cdot N}$ (°)	168	174	169	168; 167
*δ*_%_ *^a^*	96%	92%	92%	84%; 86%

*^a^* parameter *δ*_%_ corresponds to the ratio of the length of secondary interaction and the sum of van der Waals radii of two interacting atoms: *δ*_%_ $=\frac{r_{A\cdot \cdot \cdot B}}{r_{{\mathrm{vdW}}_{A}}+r_{{\mathrm{vdW}}_{B}}}\cdot 100\%$.

**Table 2:** Comparison of the sums of van der Waals radii, length (*d*), valence angles (θ) and parameters *δ*_%_ of ChBs occurring in the crystal structures of the triflates (double values refer to two interactions differing in energy).

	1-MeTfO	2-MeTfO	3-MeTfO *^a^*	4-MeTfO
$r_{{\mathrm{vdW}}_{E}}+r_{{\mathrm{vdW}}_{O}}$ (Å)	3.32	3.32	3.42	3.42
$r_{E\cdot \cdot \cdot O}$ (Å)	2.82	2.88; 3.11	2.82; 3.24	2.65; 3.03
$\theta_{N-E\cdot \cdot \cdot O}$ (°)	177	164; 158	148; 123	168; 162
*δ*_%_	85%	87%; 94%	82%; 95%	77%; 89%

*^a^* in **3-MeTfO** the parameters for Se···N are: *d* = 2.71 Å, θ = 163°, *δ*_%_ = 79%.

**Table 3:** Comparison of the sums of van der Waals radii, length (*d*), valence angles (θ) and parameters *δ*_%_ of ChBs occurring in the crystal structures of the iodides (multiple values refer to two interactions differing in energy).

	1-MeI*s ^a^*	2-MeI	3-MeI	4-MeI
$r_{{\mathrm{vdW}}_{E}}+r_{{\mathrm{vdW}}_{I}}$ (Å)	3.78	3.78	3.88	3.88
$r_{E\cdot \cdot \cdot I}$ (Å)	3.48; 3.39; 3.31	3.27	3.18; 3.61	3.067; 3.070; 3.55; 3.62
$\theta_{N-E\cdot \cdot \cdot I}$ (°)	160; 171; 170	174	174; 176	178; 176; 175; 167
*δ*_%_	92%; 90%; 88%	87%	82%; 93%	79%; 79%; 91%; 93%

*^a^* in **1-MeI** the parameters for S···N are: *d* = 3.11 Å, θ = 164°, *δ*_%_ = 93%.

**Table 4:** Global reactivity descriptors of **1**–**4** and their *N*-methyl cations [**1-Me]^+^**‒[**4-Me]^+^**.

	1	2	3	4	[1-Me]^+^	[2-Me]^+^	[3-Me]^+^	[4-Me]^+^
Dipole moment	1.99	2.45	1.36	1.85	3.23	4.66	3.59	4.16
E_HOMO_ (eV)	−6.89	−6.55	−6.75	−6.46	−11.48	−9.94	−11.30	−9.86
E_LUMO_ (eV)	−2.67	−2.34	−2.76	−2.44	−7.78	−6.93	−7.77	−6.93
ΔE_H–L gap_ (eV)	4.22	4.21	4.00	4.02	3.69	3.01	3.54	2.94
Ionization potential IP (eV)	6.89	6.55	6.75	6.46	11.48	9.94	11.30	9.86
Electron affinity EA (eV)	2.67	2.34	2.76	2.44	7.78	6.93	7.77	6.93
Hardness η (eV)	2.11	2.10	2.00	2.01	1.85	1.50	1.77	1.47
Softness ζ (eV^–1^)	0.24	0.24	0.25	0.25	0.27	0.33	0.28	0.34
Electronegativity χ (eV)	4.78	4.45	4.76	4.45	9.63	8.44	9.54	8.40
Electrophilicity index ψ (eV)	5.41	4.70	5.66	4.92	25.10	23.65	25.70	24.02

## Supplementary Materials

The following are available online at https://www.mdpi.com/1996-1944/13/21/4908/s1, Figure S1. ^1^H NMR spectrum of **1-MeTfO**, Figure S2. ^13^C NMR spectrum of **1-MeTfO**, Figure S3. ^19^F NMR spectrum of **1-MeTfO**, Figure S4. ^1^H NMR spectrum of **3-MeTfO**, Figure S5. ^13^C NMR spectrum of **3-MeTfO**, Figure S6. ^19^F NMR spectrum of **3-MeTfO**, Figure S7. ^1^H NMR spectrum of **2-MeTfO**, Figure S8. ^13^C NMR spectrum of **2-MeTfO**, Figure S9. ^19^F NMR spectrum of **2-MeTfO**, Figure S10. ^1^H NMR spectrum of **4-MeTfO**, Figure S11. ^13^C NMR spectrum of **4-MeTfO**, Figure S12. ^19^F NMR spectrum of **4-MeTfO**, Figure S13. ^1^H NMR spectrum of **1-MeI**, Figure S14. ^13^C NMR spectrum of **1-MeI**, Figure S15. ^1^H NMR spectrum of **3-MeI**, Figure S16. ^13^C NMR spectrum of **3-MeI**, Figure S17. ^1^H NMR spectrum of **2-MeI**, Figure S18. ^13^C NMR spectrum of **2-MeI**, Figure S19. ^1^H NMR spectrum of **4-MeI**, Figure S20. ^13^C NMR spectrum of **4-MeI**, Figure S21. The most important molecular orbitals taking part in the electronic transitions for the neutral form of investigated molecules as calculated by the TD-DFT/B3LYP/6-31++G(d,p) methods (isosurface value equal to 0.04 a.u.^−3/2^), Figure S22. The most important molecular orbitals taking part in the electronic transitions for the cationic form of investigated molecules as calculated by the TD-DFT/B3LYP/6-31++G(d,p) methods (isosurface value equal to 0.04 a.u.^−3/2^), Figure S23. Fragment of the catemer formed by molecules of **1** (a) and **3** (b). Figure S24. (a) Crystal packing of **2** viewed along the [100] direction; (b) the **2**_2_ dimer, Figure S25. (a) Crystal packing of **2** viewed along the [100] direction; **(b)** the two different **4**_2_ dimers, Figure S26. (a) Crystal packing of **2-MeTfO** viewed along the [100] direction; **(b)** the blocks of cations of **2-MeTfO** viewed along *c** axis. Figure S27. (a) Crystal packing of **4-MeTfO** viewed along the [100] direction; (b) the blocks of cations of **4-MeTfO** viewed along *c** axis. Figure S28. Crystal packing of **1-MeI** viewed along the *a* axis, Figure S29. (a) Crystal packing of **2-MeI** viewed along the *a* axis; (b) view of the polymeric chains, Figure S30. Crystal packing of **4-MeI** viewed along the *a* axis, Table S1. Selected crystallographic data, Table S2. The most important (oscillator strength > 0.03) electronic transitions of the neutral form of investigated compounds as calculated by the TD-DFT/B3LYP-D3/6-31++G(d,p) method in methanol, Table S3. The most important (oscillator strength > 0.03) electronic transitions of the cationic form of investigated compounds as calculated by the TD-DFT/B3LYP-D3/6-31++G(d,p) method in methanol.
