# Supplementary material for: Effect of Aromatic System Expansion on Crystal Structures of 1,2,5-Thia- and 1,2,5-Selenadiazoles and Their Quaternary Salts: Synthesis, Structure, and Spectroscopic Properties
Source: Materials (Basel). 2020 Oct 31;13(21):4908. doi: 10.3390/ma13214908 (PMC7663733; doi:10.3390/ma13214908)
Supplement: Supplementary file 1 [file materials-13-04908-s001.pdf]

materials

Supplementary Materials: Effect of Aromatic System Expansion on Crystal Structures of 1,2,5-Thia- and 1,2,5-Selenadiazoles and Their Quaternary Salts: Synthesis, Structure, and Spectroscopic Properties

Jan Alfuth [1], Beata Zadykowicz [2], Artur Sikorski [3], Tadeusz Połoński [1], Katarzyna Eichstaedt [1] and Teresa Olszewska [1,*]

[1] Department of Organic Chemistry, Gdańsk University of Technology, 80-233 Gdańsk, Poland; jan.alfuth@pg.edu.pl (J.A.), tadeusz.polonski@pg.edu.pl (T.P.), kat.eichstaedt@gmail.com (K.E.)
[2] Luminescence Research Group, Faculty of Chemistry, University of Gdańsk, 80-308 Gdańsk, Poland; beata.zadykowicz@ug.edu.pl
[3] Laboratory of Crystallochemistry, Faculty of Chemistry, University of Gdańsk, 80-308 Gdańsk, Poland; artur.sikorski@ug.edu.pl
* Correspondence: teresa.olszewska@pg.edu.pl; Tel.: +48-58-347-14-25

1. Experimental Procedures

1.1. General Procedure for Preparation Of N-Methylphenanthro[9,10-c][1,2,5]chalcogenadiazolium Triflates

Methyl triflate (2 mmol) was added slowly to a solution of a 2,1,3-benzochalcogenadiazole or phenanthro[9,10-*c*][1,2,5]chalcogenadiazole (1 mmol) in 30 mL of anhydrous 1,2-dichloroethane at 40 °C. The mixture was then stirred for 24–48 h at 60 °C. Diethyl ether was added to crush out as much product as possible. The precipitate was filtered under reduced pressure, washed with fresh portion of diethyl ether and dried.

1.1.1. *N*-Methyl-2,1,3-benzothiadiazolium Triflate (**1-MeTfO**)

Yield: 88%. The X-ray quality crystals were obtained from a mixture of CH_3CN and $PhCH_3$; pale yellow needles, mp 87–89 °C (lit. 88–90 °C [1]). ^1H NMR (400 MHz, DMSO-d_6): δ 8.36 (d, *J* = 8.9 Hz, 1H), 8.30–8.21 (m, 2H), 8.03 (ddd, *J* = 8.9; 6.2; 1.5 Hz, 1H), 4.67 (s, 3H). ^{13}C NMR (100 MHz, DMSO-d_6): δ 152.47, 146.66, 137.35, 132.32, 123.97, 121.13 (q, *J* = 322.4 Hz), 115.75, 37.84. ^{19}F NMR (376 MHz, DMSO-d_6): δ – 77.75.

1.1.2. *N*-Methyl-2,1,3-benzoselenadiazolium Triflate (**3-MeTfO**)

Yield: 85%. The X-ray quality crystals were obtained from dichloroethane; yellow needles, mp 153–155 °C (lit. 157–158 °C [1]). ^1H NMR (400 MHz, DMSO-d_6): δ 8.07–7.92 (m, 3H), 7.79 (ddd, *J* = 9.0; 6.0; 1.6 Hz, 1H), 4.58 (s, 3H). ^{13}C NMR (100 MHz, DMSO-d_6): δ 156.81, 151.41, 137.13, 130.59, 125.52, 121.14 (q, *J* = 322.3 Hz), 116.83, 38.80. ^{19}F NMR (376 MHz, DMSO-d_6): δ –77.76.

1.1.3. *N*-Methylphenanthro[9,10-*c*][1,2,5]thiadiazolium Triflate (**2-MeTfO**)

Yield: 68%. The X-ray quality crystals were obtained using vapor diffusion technique form acetone-MeOH/Et_2O; yellow needles, mp 228–232 °C. ^1H NMR (400 MHz, DMSO-d_6): δ 9.02 (d, *J* = 8.4 Hz, 1H), 8.92 (d, *J* = 8.4 Hz, 1H), 8.89 (d, *J* = 8.2 Hz, 1H), 8.69 (dd, *J* = 7.8; 1.3 Hz, 1H), 8.18–8.11 (m, 1H), 8.03–7.89 (m, 3H), 4.96 (s, 3H). ^{13}C NMR (100 MHz, DMSO-d_6): δ 153.09, 146.63, 134.34, 134.24, 132.49, 131.47, 130.48, 129.81, 128.44, 125.83, 125.43, 125.01, 124.65, 121.41, 121.16 (q, *J* = 322.4 Hz), 41.52. ^{19}F NMR (376 MHz, DMSO-d_6): δ –77.73.

1.1.4. *N*-Methylphenanthro[9,10-*c*][1,2,5]selenadiazolium Triflate (**4-MeTfO**)

Yield: 58%. The X-ray quality crystals were obtained using vapor diffusion technique form acetone-MeOH/Et_2O; yellow needles, mp 248–250 °C. ^{1}H NMR (400 MHz, DMSO-d_6): δ 8.87 (d, *J* = 8.6 Hz, 2H), 8.73 (d, *J* = 8.0 Hz, 1H), 8.60 (dd, *J* = 7.9; 1.3 Hz, 1H), 8.11–8.01 (m, 1H), 7.94–7.78 (m, 3H), 4.84 (s, 3H). ^{13}C NMR (100 MHz, DMSO-d_6): δ 155.61, 150.29, 134.44, 134.10, 131.87, 130.97, 130.11, 129.45, 129.42, 127.36, 125.80, 125.51, 124.52, 123.78, 121.16 (q, *J* = 322.5 Hz), 42.53. ^{19}F NMR (376 MHz, DMSO-d_6): δ – 77.72.

1.2. General Procedure for Preparation of N-Methyl-2,1,3-benzochalcogenadiazolium Iodides

To a solution of a *N*-methyl-2,1,3-benzochalcogenadiazolium triflate (1 mmol) in 10 mL of methanol tetrabutylammonium iodide (2 mmol) dissolved in 5 mL of methanol was added dropwise. Toluene was added to crush out as much product as possible. Resulting deep red precipitate was filtered under reduced pressure, washed with diethyl ether and dried.

1.2.1. *N*-Methyl-2,1,3-benzothiadiazolium Triflate (**1-MeI**)

Yield: 83%. The X-ray quality crystals were obtained from a mixture of MeOH and $PhCH_3$; purple needles, mp 145–147 °C (lit. 149–150 °C [2]). ^{1}H NMR (400 MHz, DMSO-d_6): δ 8.36 (d, *J* = 8.9 Hz, 1H), 8.31–8.20 (m, 2H), 8.03 (ddd, *J* = 8.8, 6.4, 1.2 Hz, 1H), 4.66 (s, 3H). ^{13}C NMR (100 MHz, DMSO-d_6): δ 152.39, 146.54, 137.24, 132.26, 123.97, 115.79, 38.06.

1.2.2. *N*-Methyl-2,1,3-benzoselenadiazolium Triflate (**3-MeI**)

Yield: 89%. The X-ray quality crystals were obtained from CH_3CN; brick-red needles, mp 174–175 °C (lit. 171–172 °C [2]). ^{1}H NMR (400 MHz, DMSO-d_6): δ 8.03–7.91 (m, 1H), 7.76 (ddd, *J* = 8.9, 6.1, 1.3 Hz, 1H), 4.54 (s, 1H). ^{13}C NMR (100 MHz, DMSO-d_6): δ 156.87, 151.08, 136.94, 130.33, 125.50, 117.03, 38.91.

1.3. General Procedure for Preparation of N-Methylphenanthro[9,10-c][1,2,5]chalcogenadiazolium Iodides

To a solution of a *N*-methylphenanthro[9,10-*c*][1,2,5]chalcogenadiazolium triflate (1 mmol) in 25 mL of acetone NaI (2 mmol) dissolved in 5 mL of acetone was added dropwise. Resulting red precipitate was filtered under reduced pressure, washed with diethyl ether and dried.

1.3.1. *N*-Methylphenanthro[9,10-*c*][1,2,5]thiadiazolium Iodide (**2-MeI**)

Yield: 93%. The X-ray quality crystals were obtained using vapor diffusion technique form acetone-MeOH/Et_2O; red needles, mp 165–166 °C. ^{1}H NMR (400 MHz, DMSO-d_6): δ 9.04 (d, *J* = 8.0 Hz, 1H), 8.94 (d, *J* = 8.0 Hz, 1H), 8.91 (d, *J* = 8.2 Hz, 1H), 8.71 (dd, *J* = 7.8; 1.3 Hz, 1H), 8.19–8.11 (m, 1H), 8.05–7.90 (m, 3H), 4.97 (s, 3H). ^{13}C NMR (100 MHz, DMSO-d_6): δ 153.13, 146.56, 134.30, 132.45, 131.45, 130.70, 129.80, 129.09, 128.42, 125.90, 125.39, 124.98, 124.67, 121.39, 41.60.

1.3.2. *N*-Methylphenanthro[9,10-*c*][1,2,5]selenadiazolium Iodide (**4-MeI**)

Yield: 55%. The X-ray quality crystals were obtained from DMF; red columns, mp 189 °C (decomp.). ^{1}H NMR (400 MHz, DMSO-d_6): δ 8.89 (d, *J* = 7.6 Hz, 1H), 8.88 (d, *J* = 7.7 Hz, 1H), 8.74 (d, *J* = 8.0 Hz, 1H), 8.62 (dd, *J* = 7.9; 1.3 Hz, 1H), 8.11–8.02 (m, 1H), 7.94–7.78 (m, 3H), 4.83 (s, 3H). ^{13}C NMR (100 MHz, DMSO-d_6): δ 155.62, 150.15, 134.46, 134.08, 131.86, 130.95, 130.14, 129.53, 129.44, 127.49, 125.86, 125.56, 124.57, 123.94, 42.49.

2. NMR Spectra

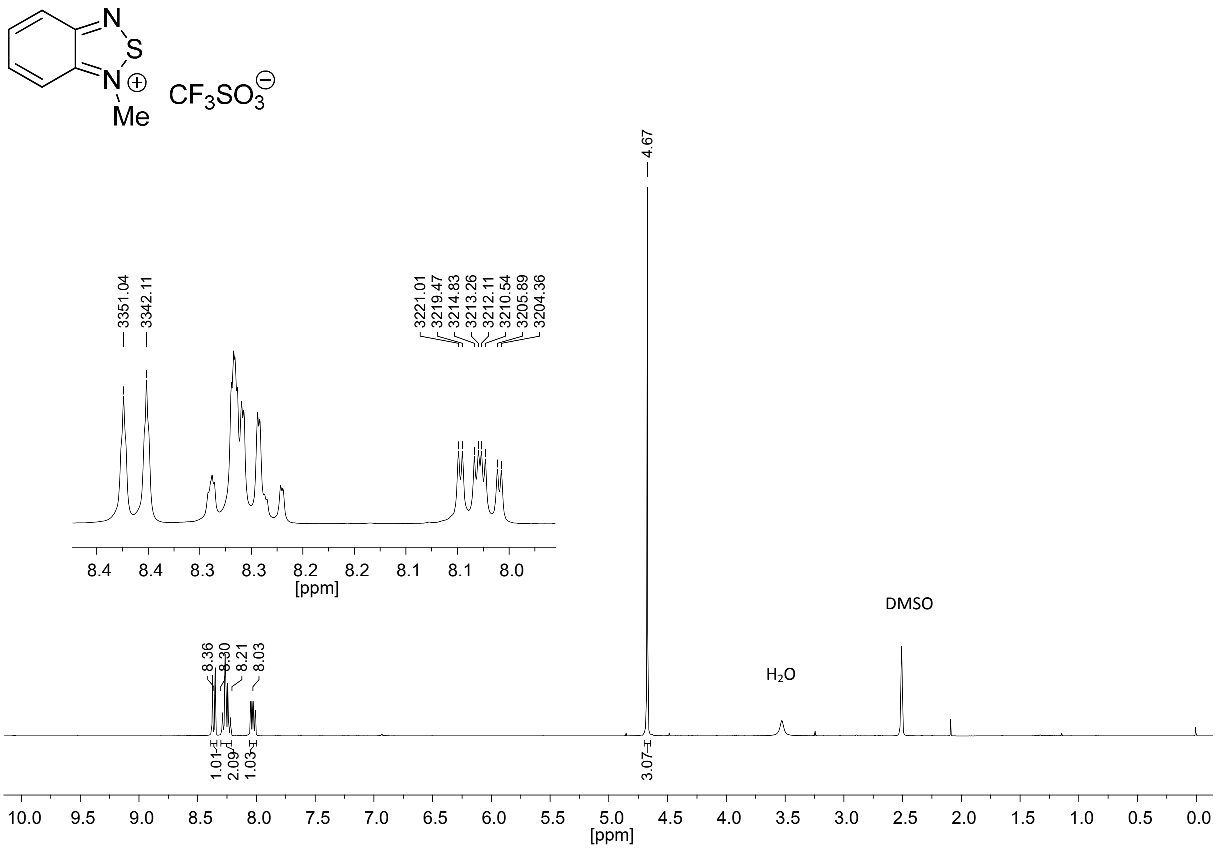

Figure S1. ^{1}H NMR spectrum of **1-MeTfO**.

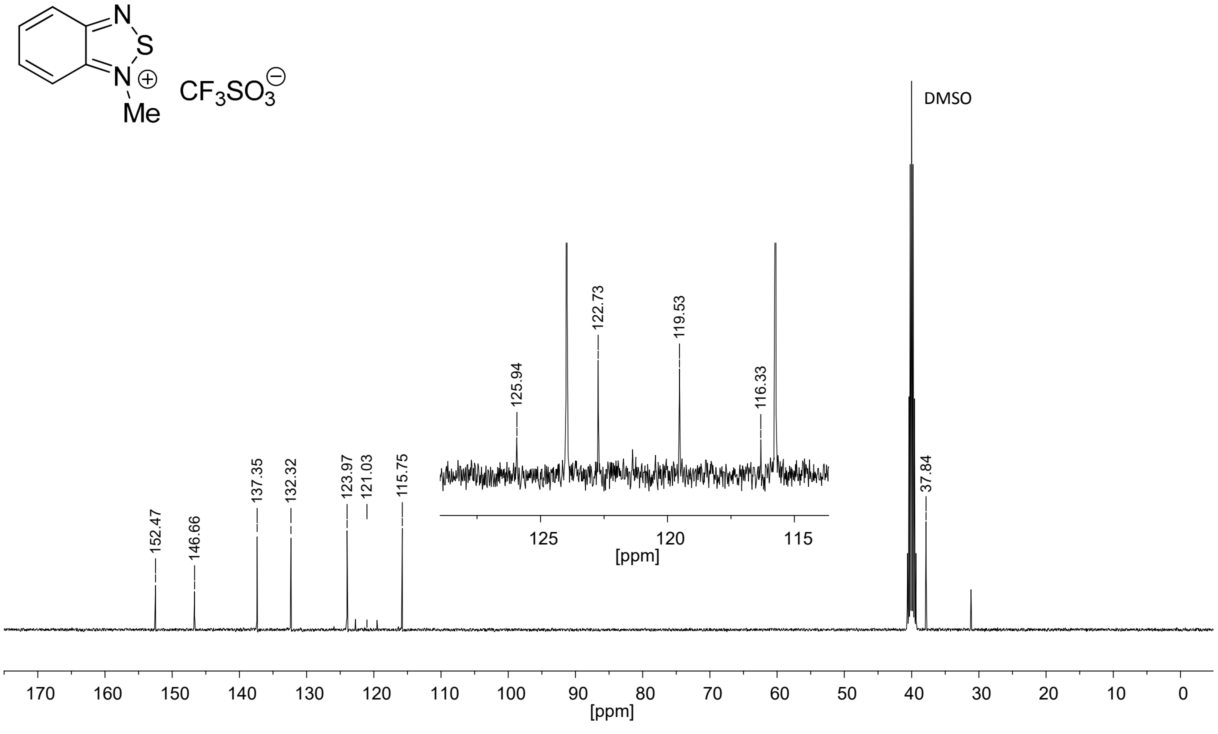

Figure S2. ^{13}C NMR spectrum of **1-MeTfO**.

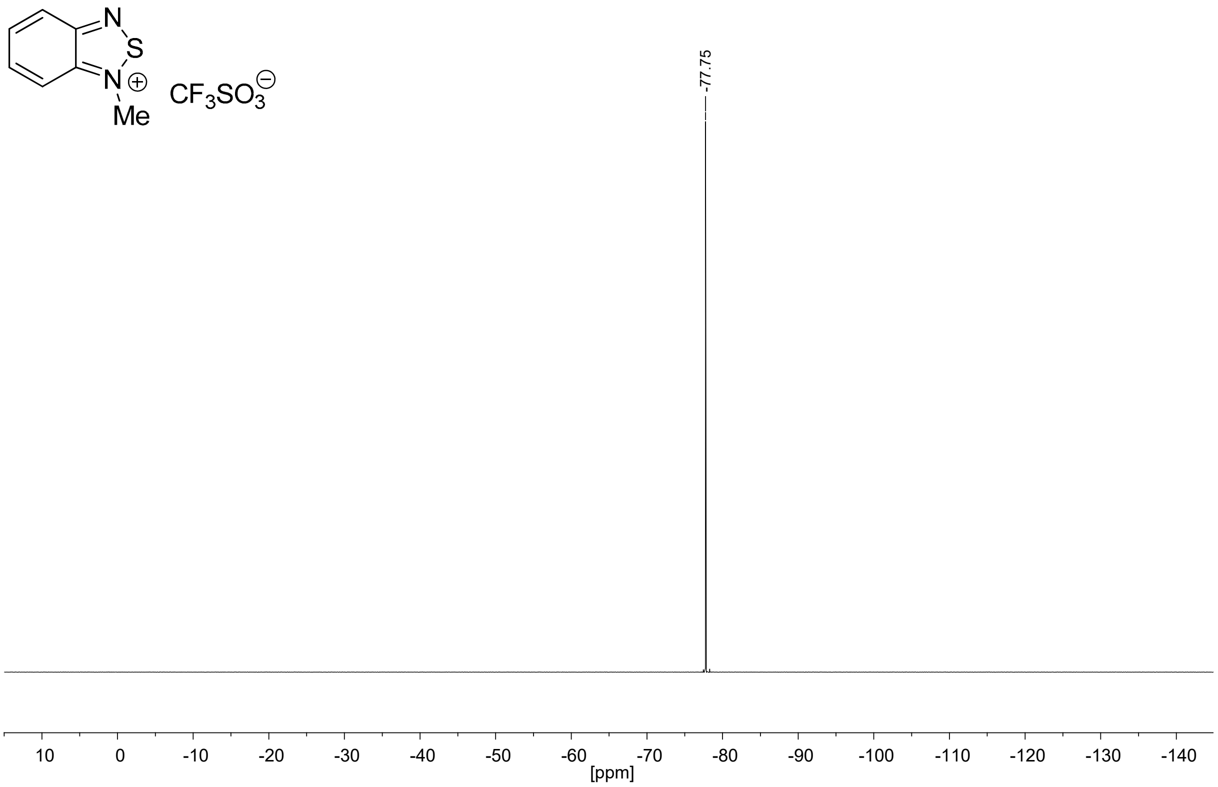

Figure S3. ^{19}F NMR spectrum of **1-MeTfO**.

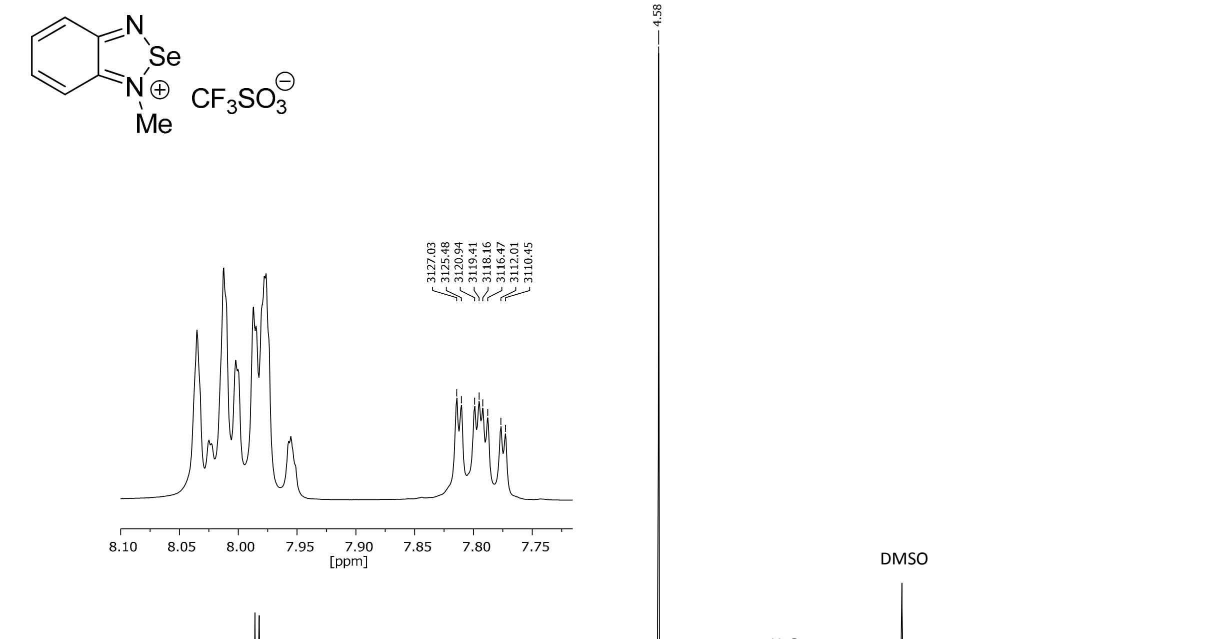

Figure S4. ^{1}H NMR spectrum of **3-MeTfO**.

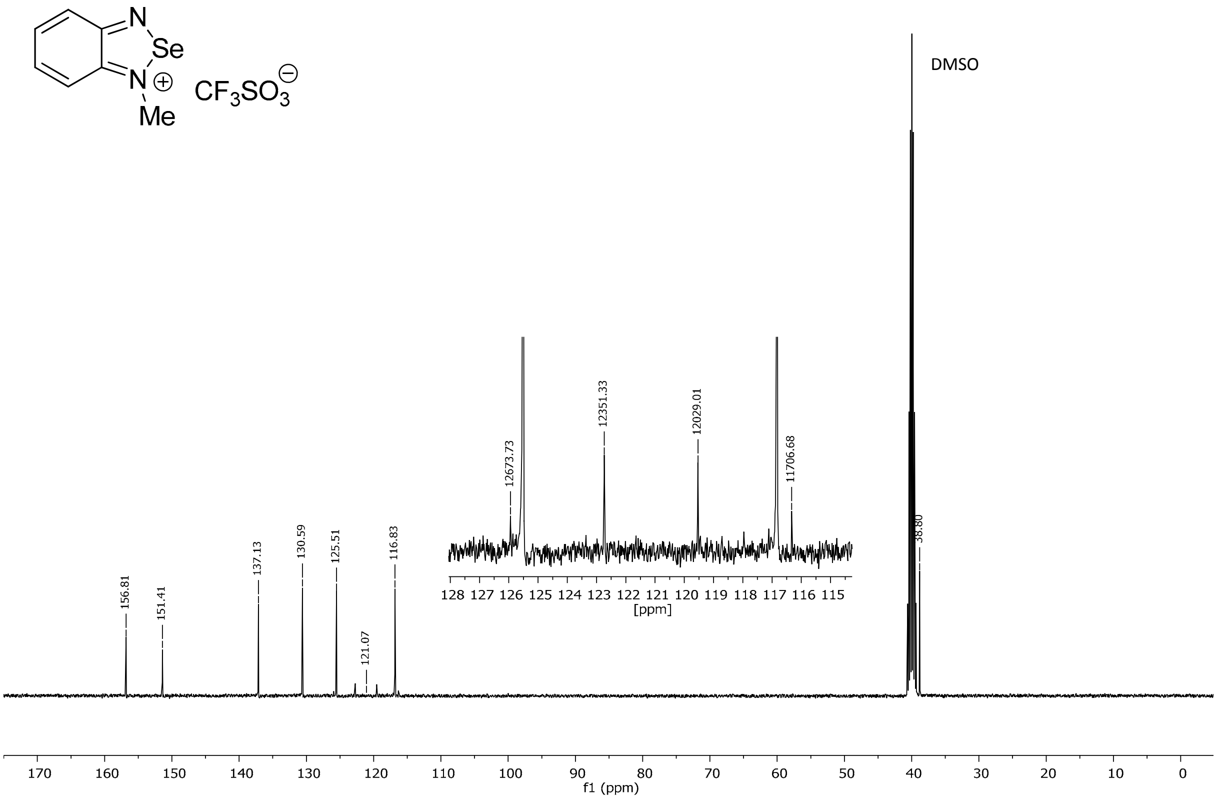

Figure S5. ^{13}C NMR spectrum of **3-MeTfO**.

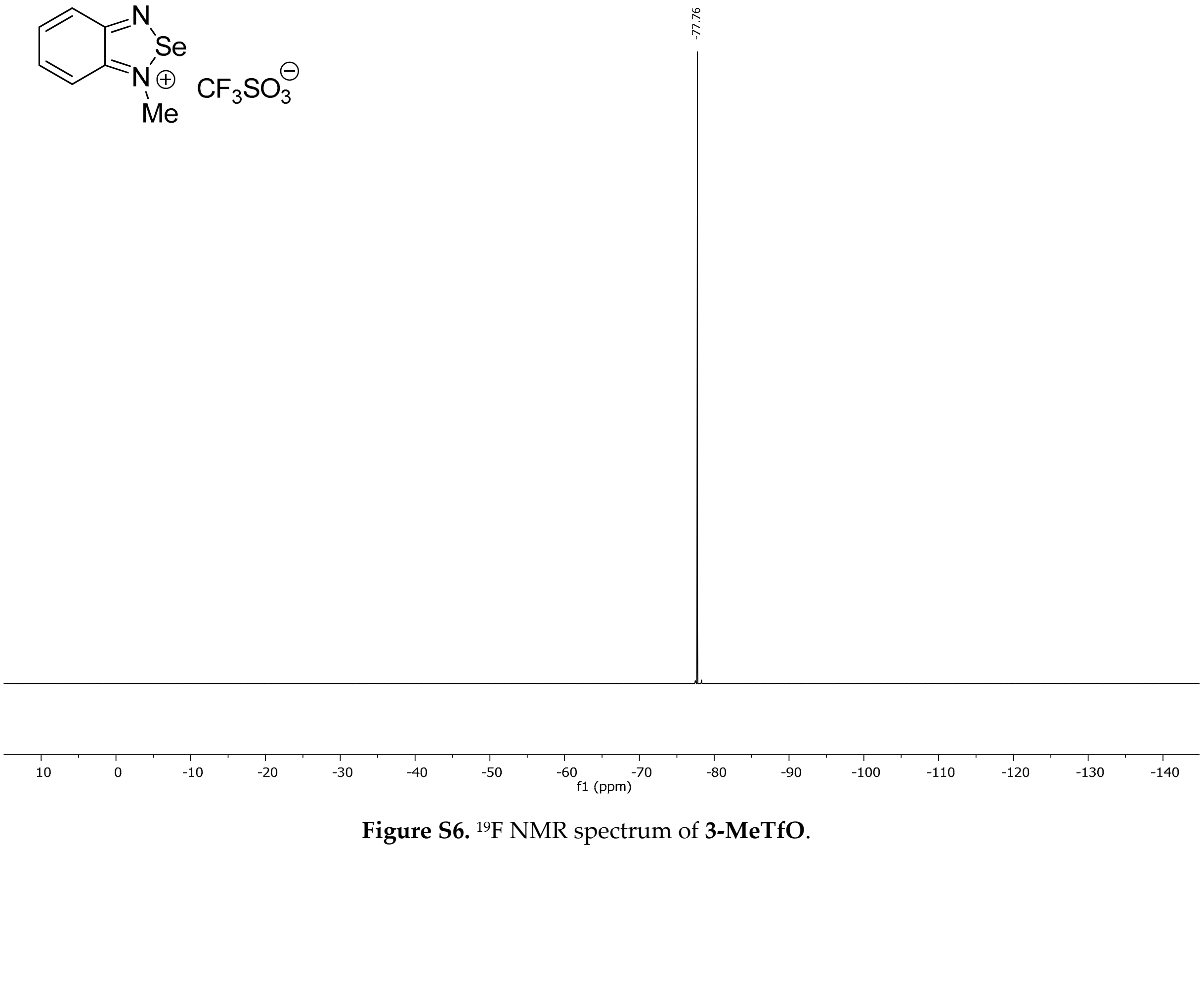

Figure S6. ^{19}F NMR spectrum of **3-MeTfO**.

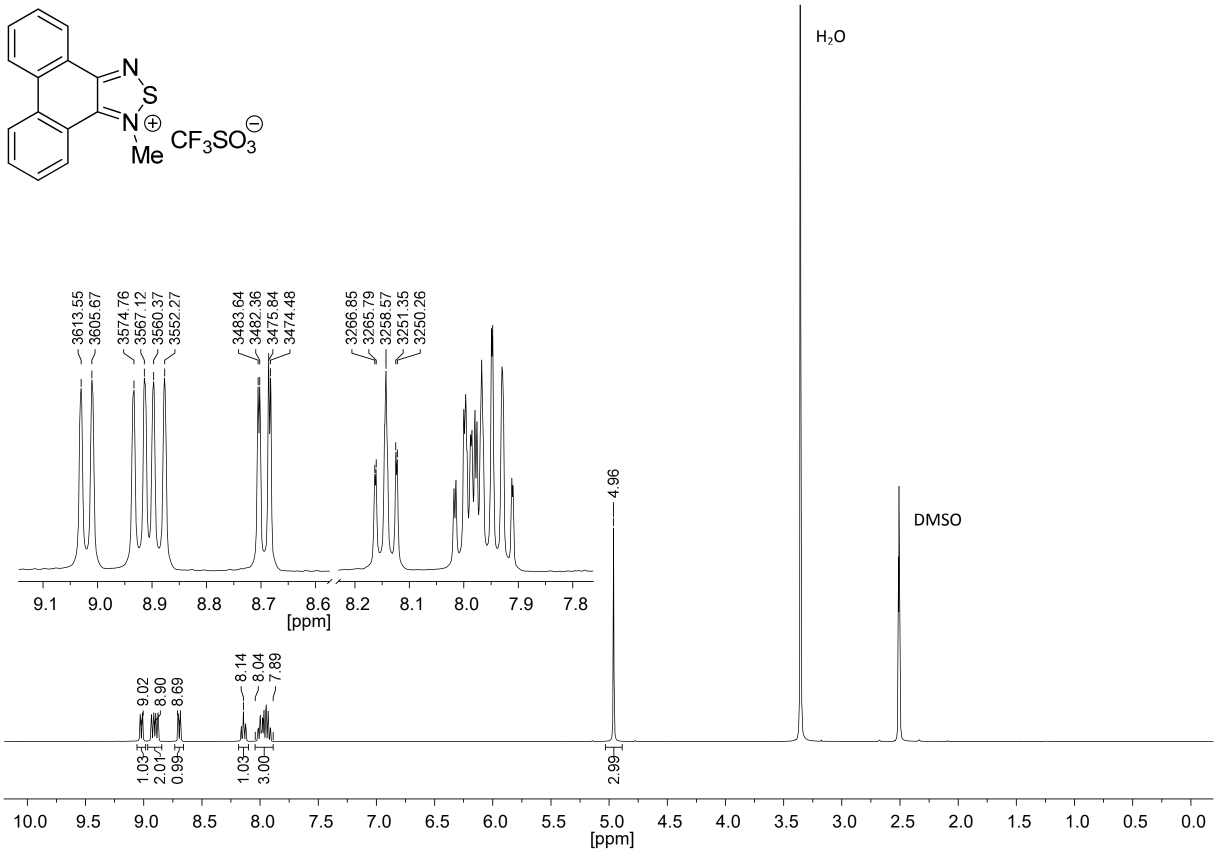

Figure S7. ^{1}H NMR spectrum of **2-MeTfO**.

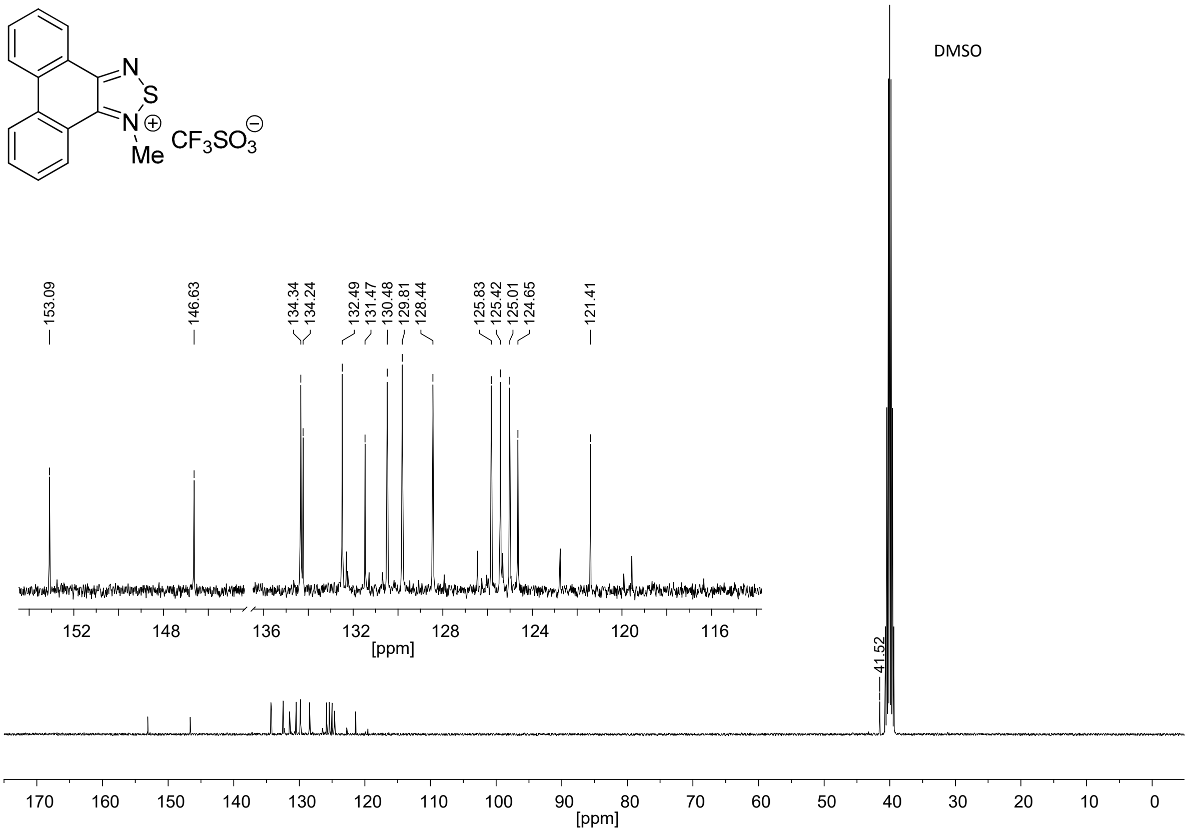

Figure S8. ^{13}C NMR spectrum of **2-MeTfO**.

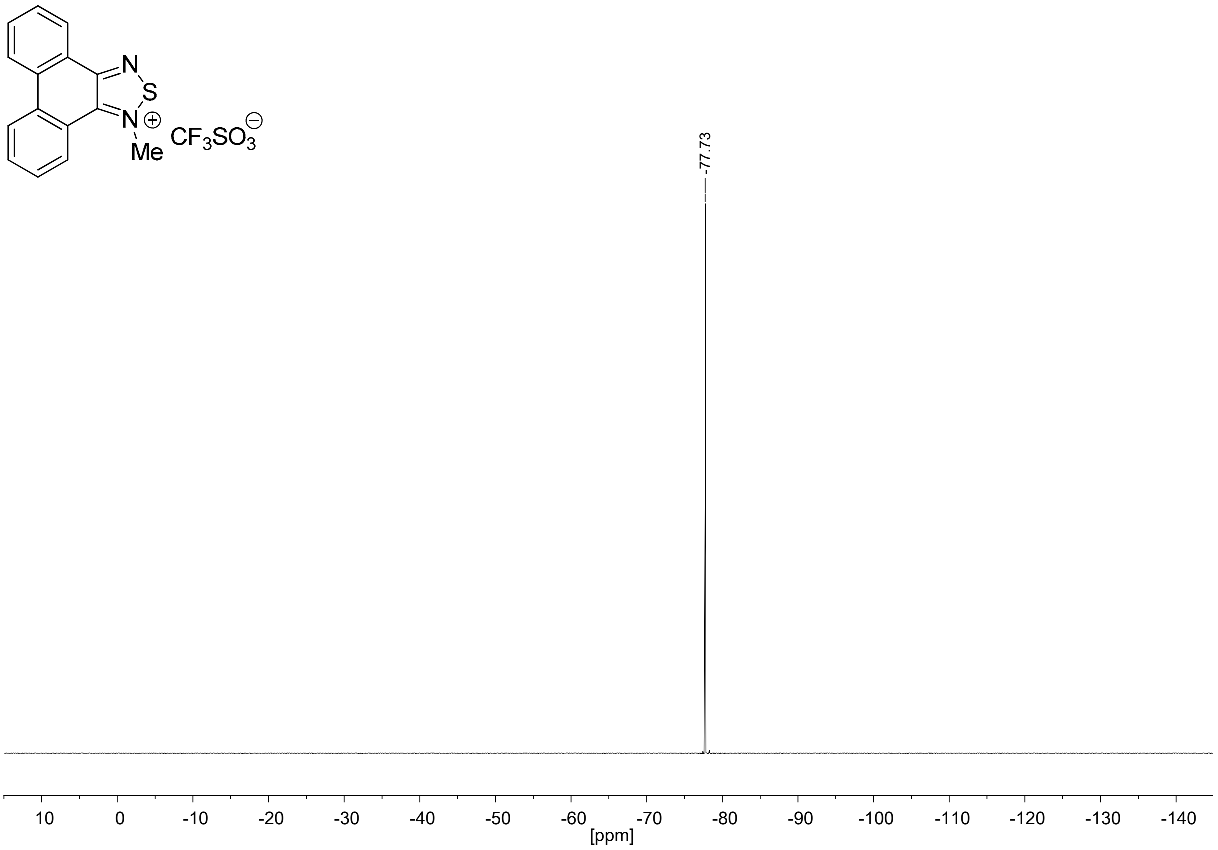

Figure S9. ^{19}F NMR spectrum of **2-MeTfO**.

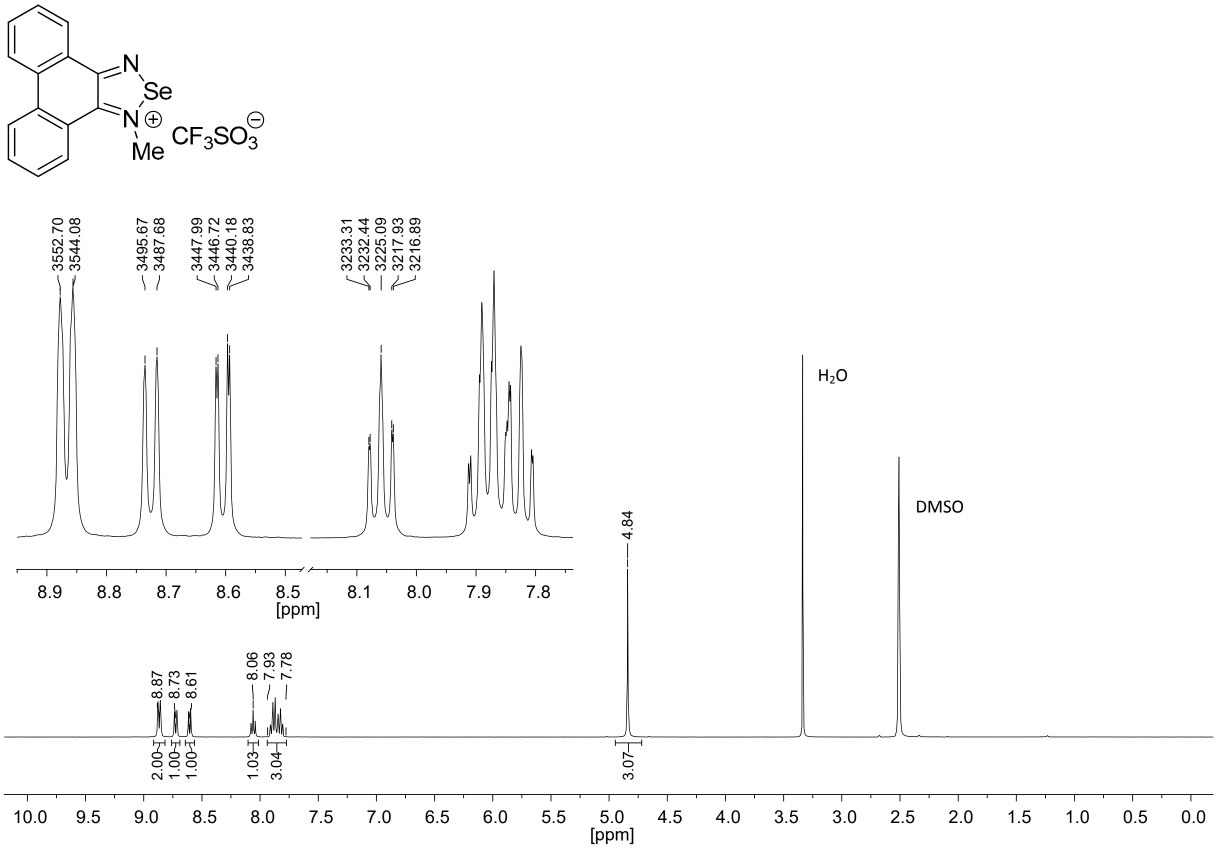

Figure S10. ^{1}H NMR spectrum of **4-MeTfO**.

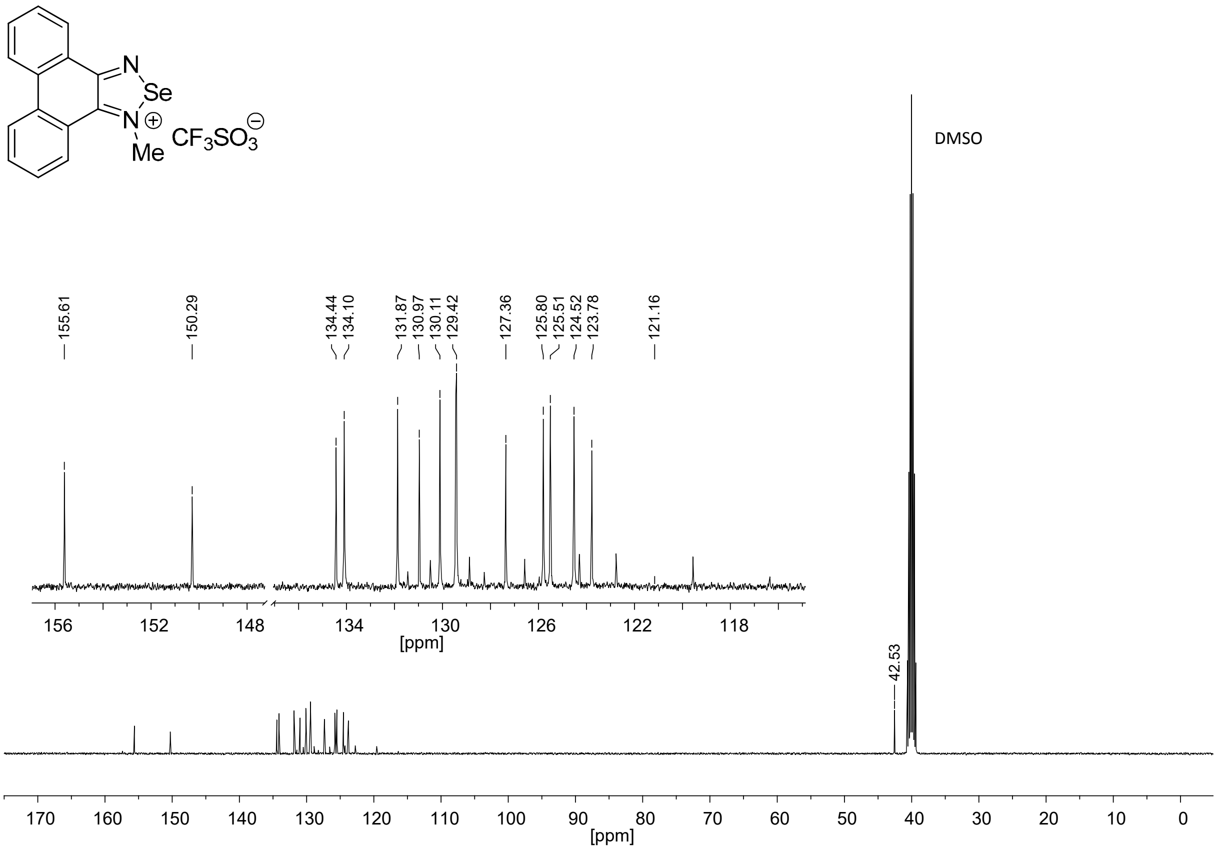

Figure S11. ^{13}C NMR spectrum of **4-MeTfO**.

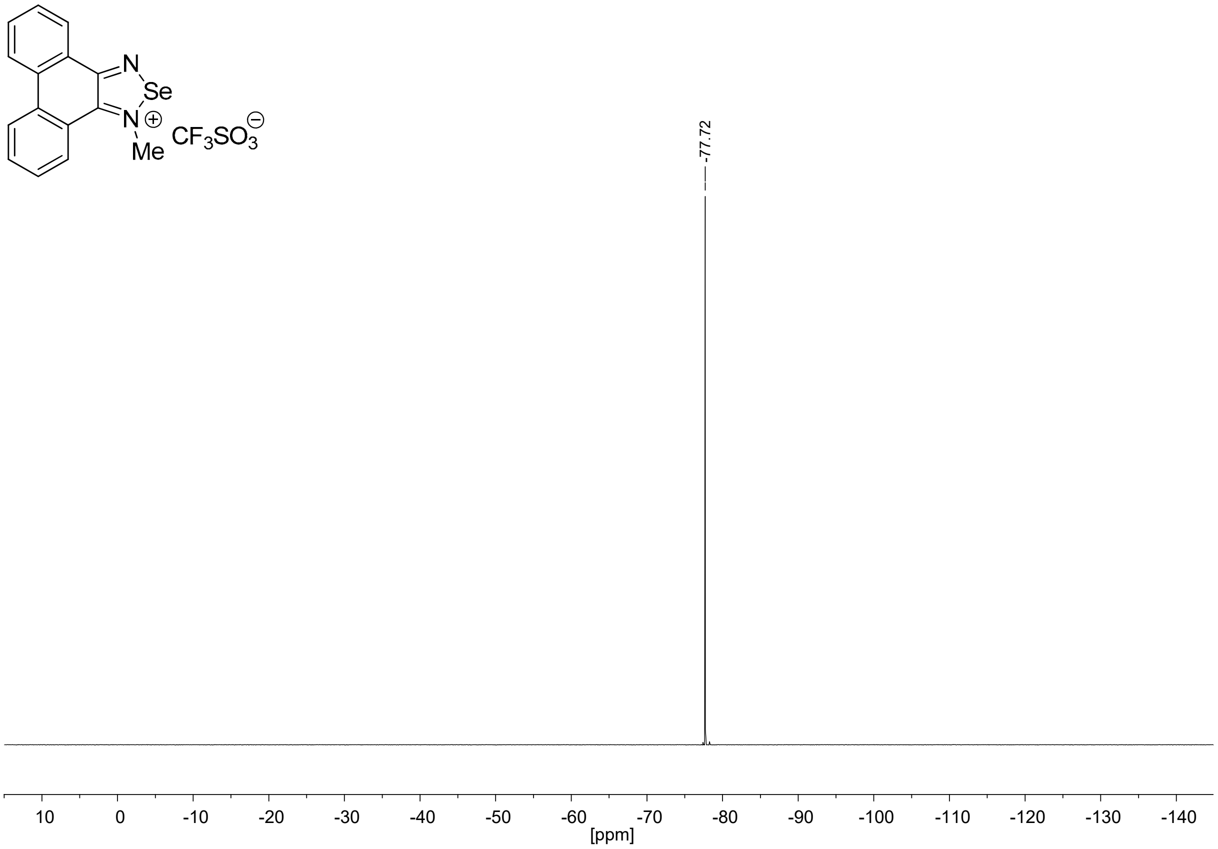

Figure S12. ^{19}F NMR spectrum of **4-MeTfO**.

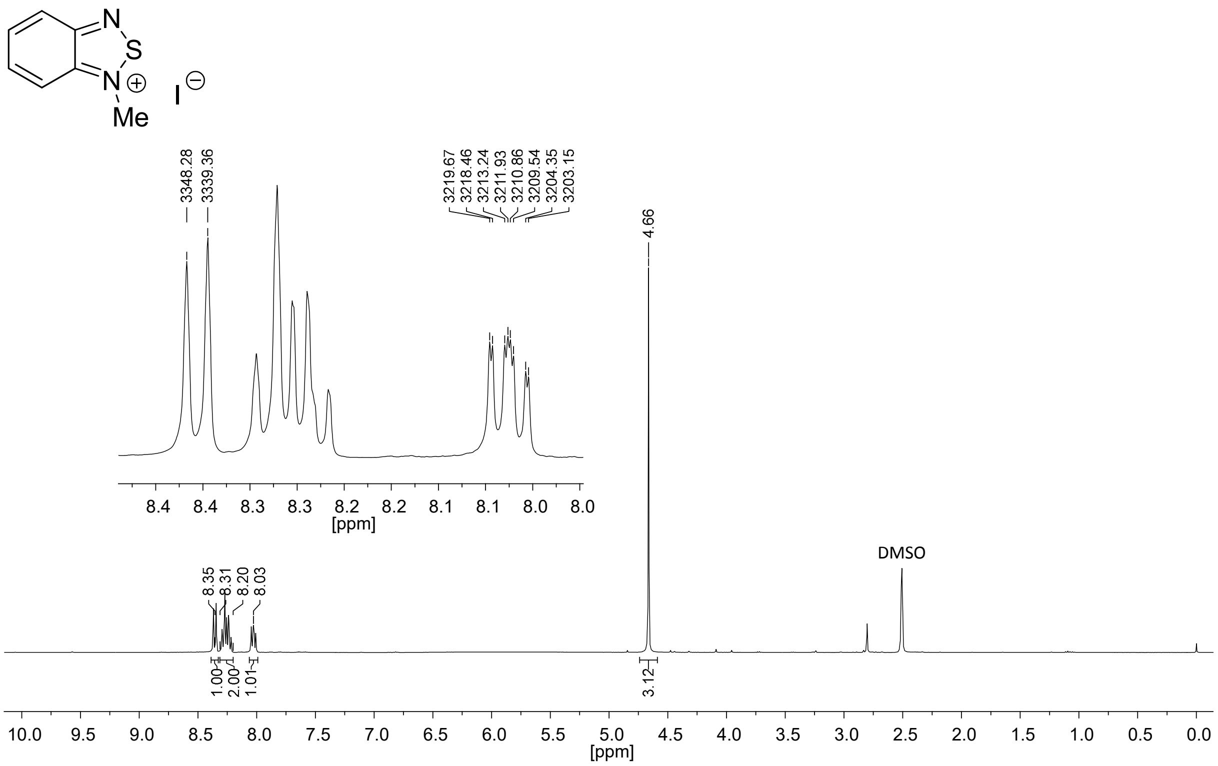

Figure S13. ^{1}H NMR spectrum of **1-MeI**.

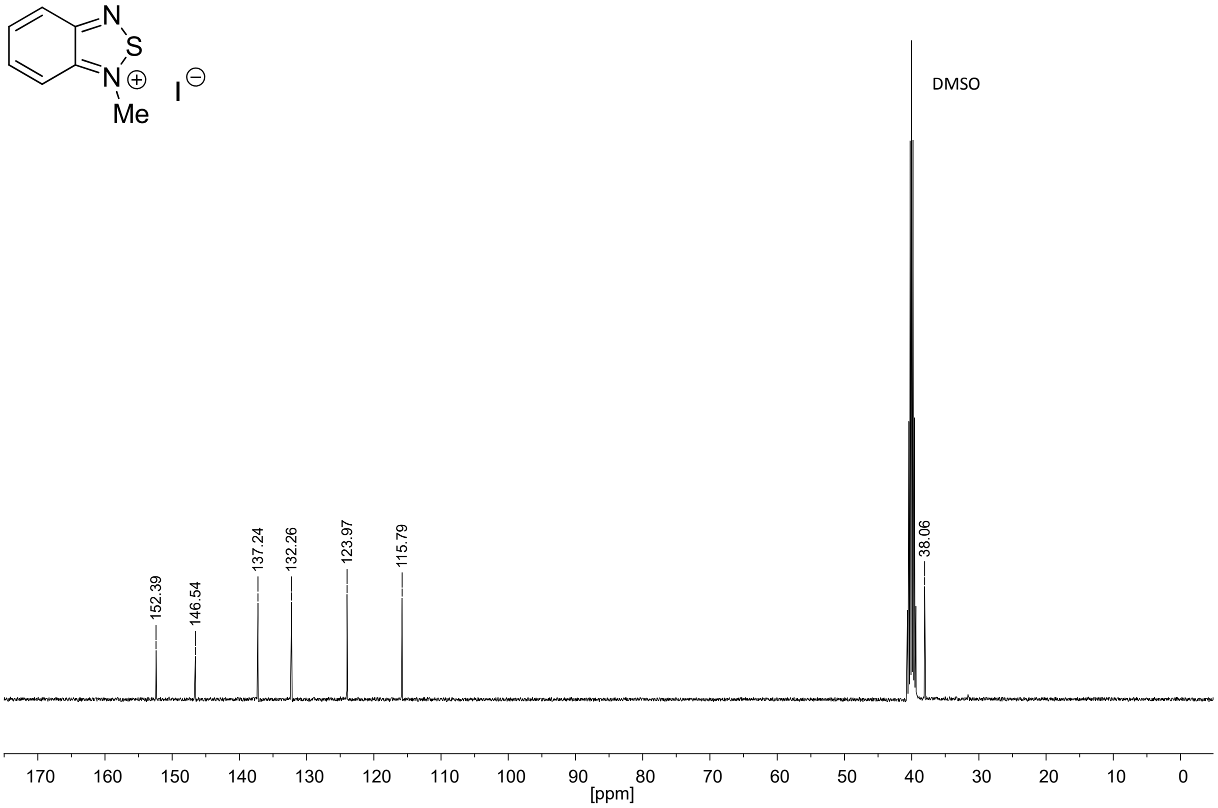

Figure S14. ^{13}C NMR spectrum of **1-MeI**.

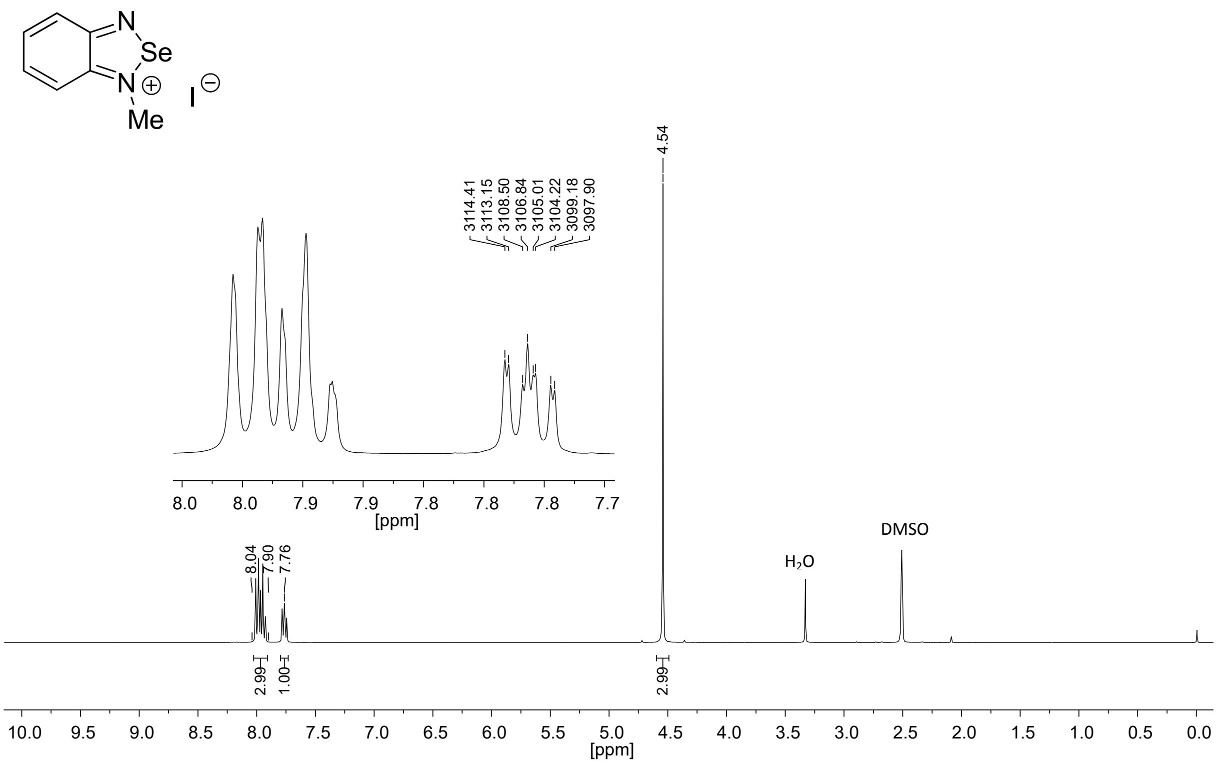

Figure S15. ^{1}H NMR spectrum of **3-MeI**.

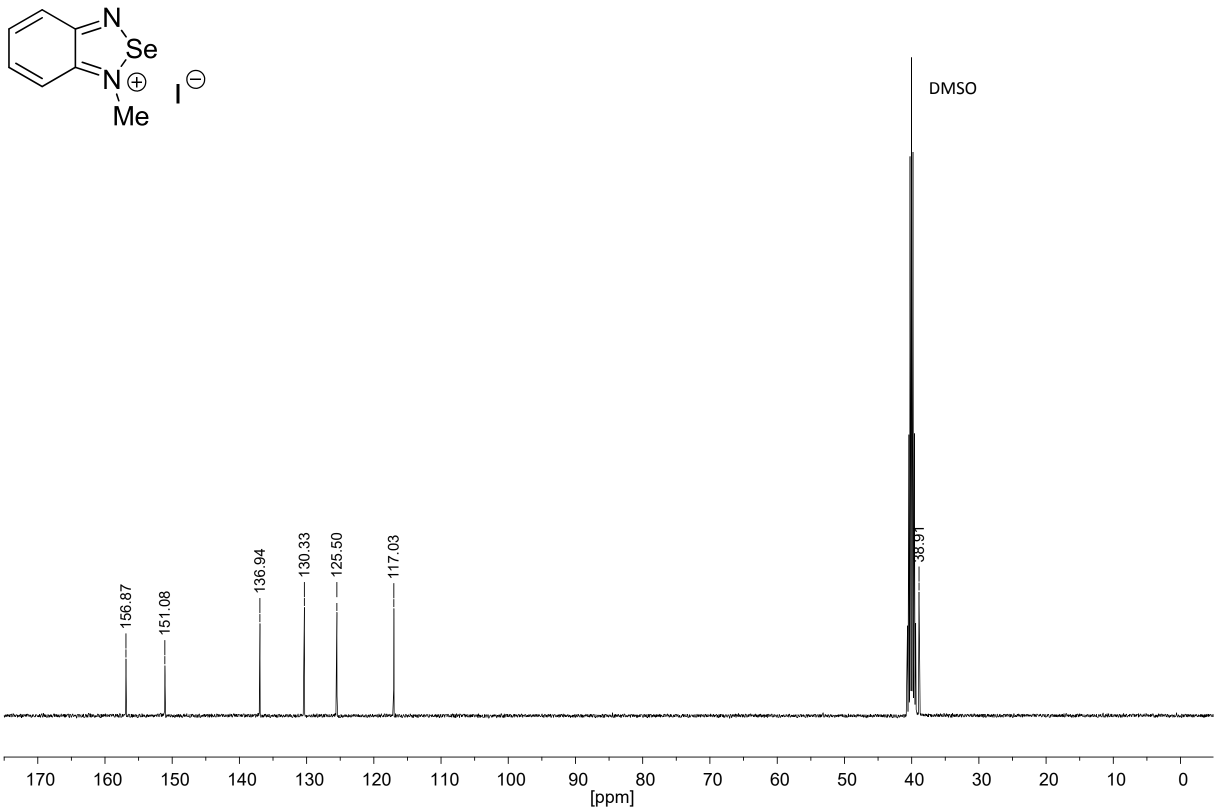

Figure S16. ^{13}C NMR spectrum of **3-MeI**.

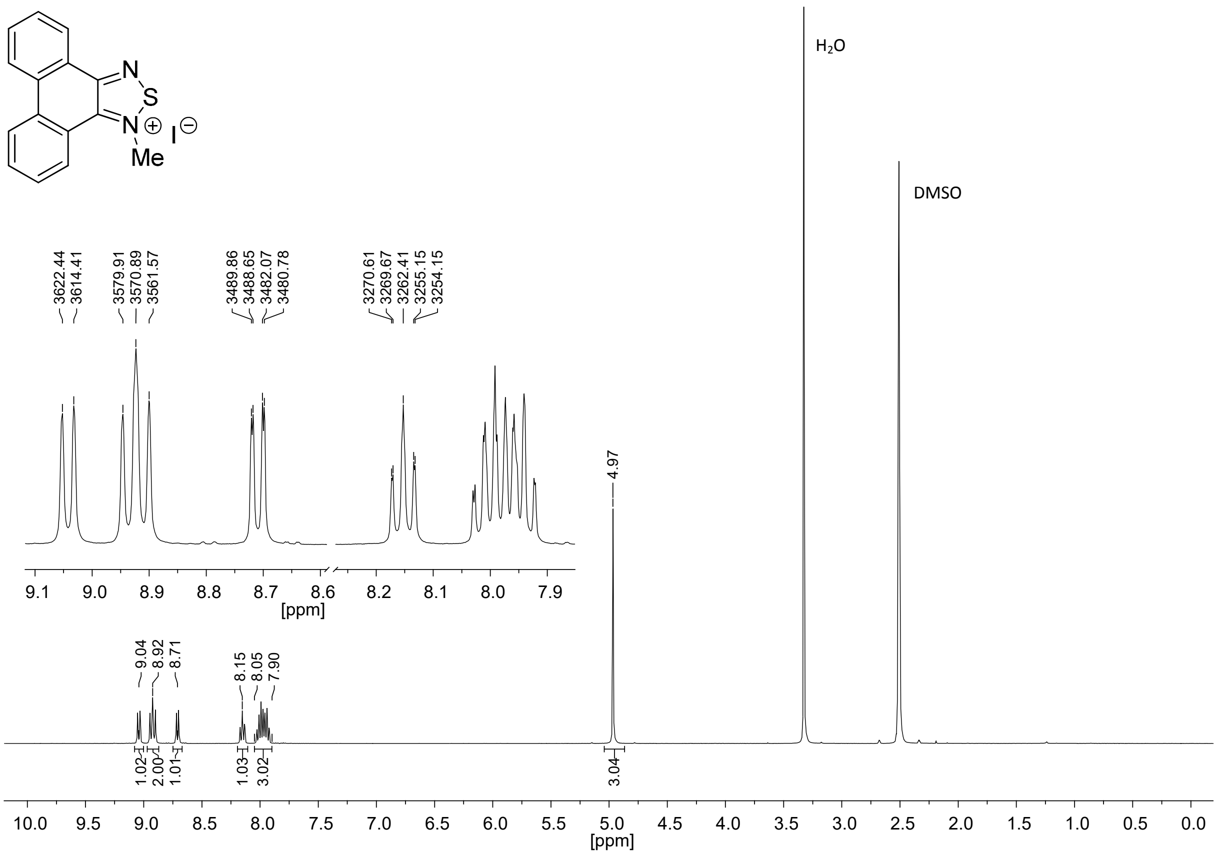

Figure S17. ^{1}H NMR spectrum of **2-MeI**.

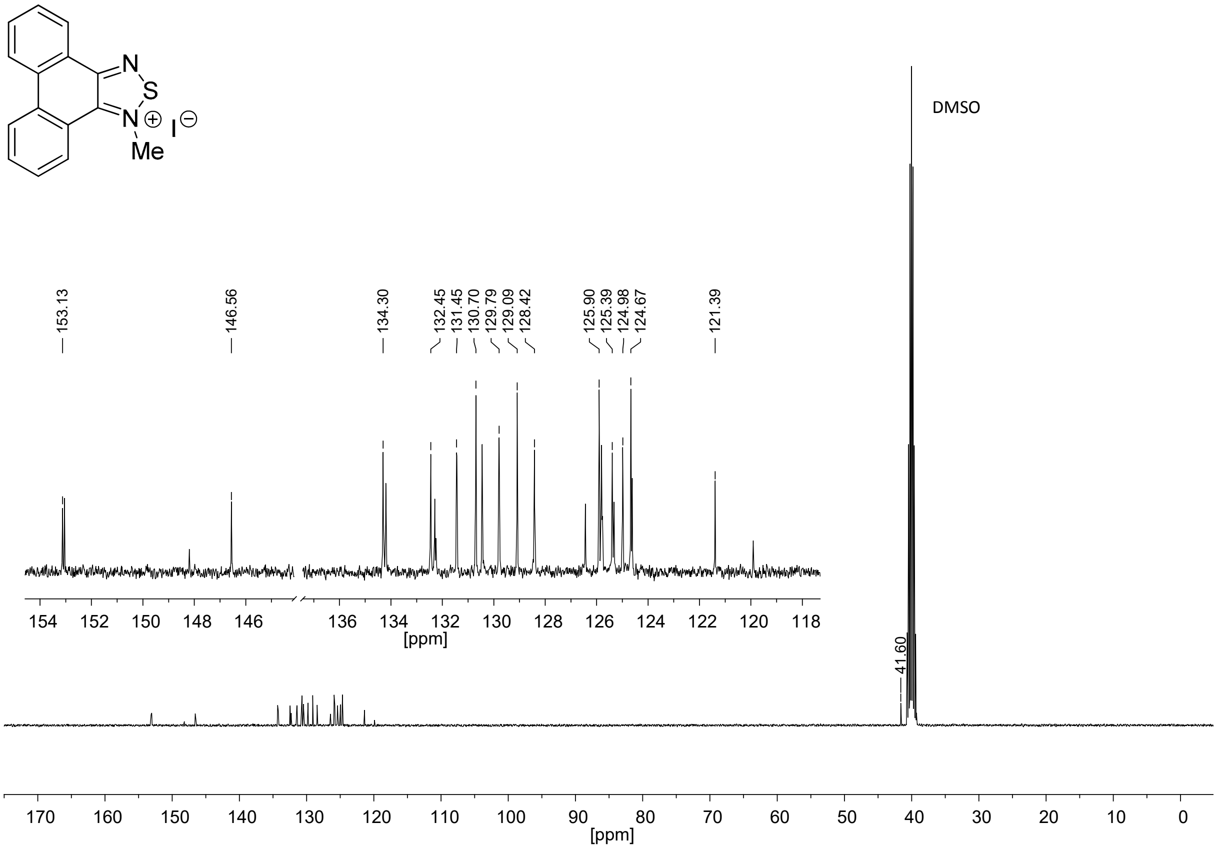

Figure S18. ^{13}C NMR spectrum of **2-MeI**.

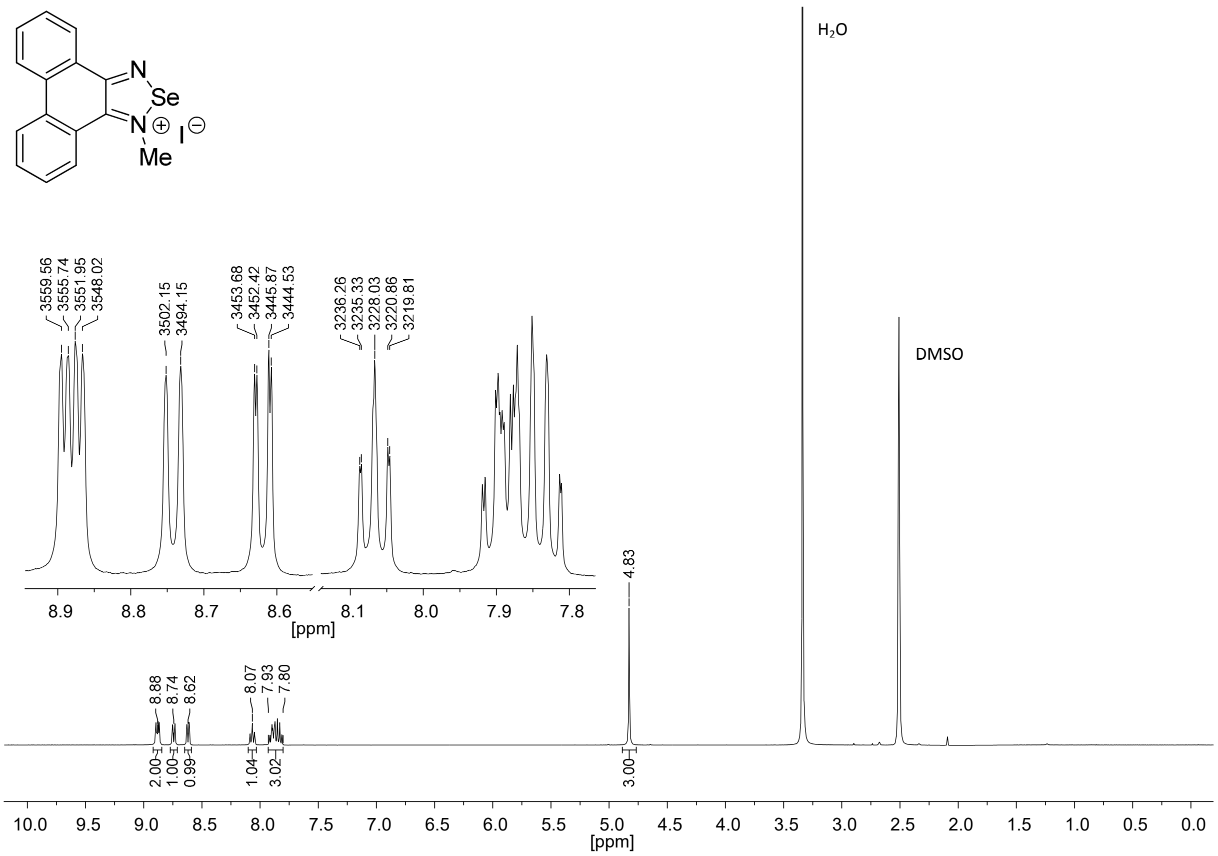

Figure S19. ^{1}H NMR spectrum of **4-MeI**.

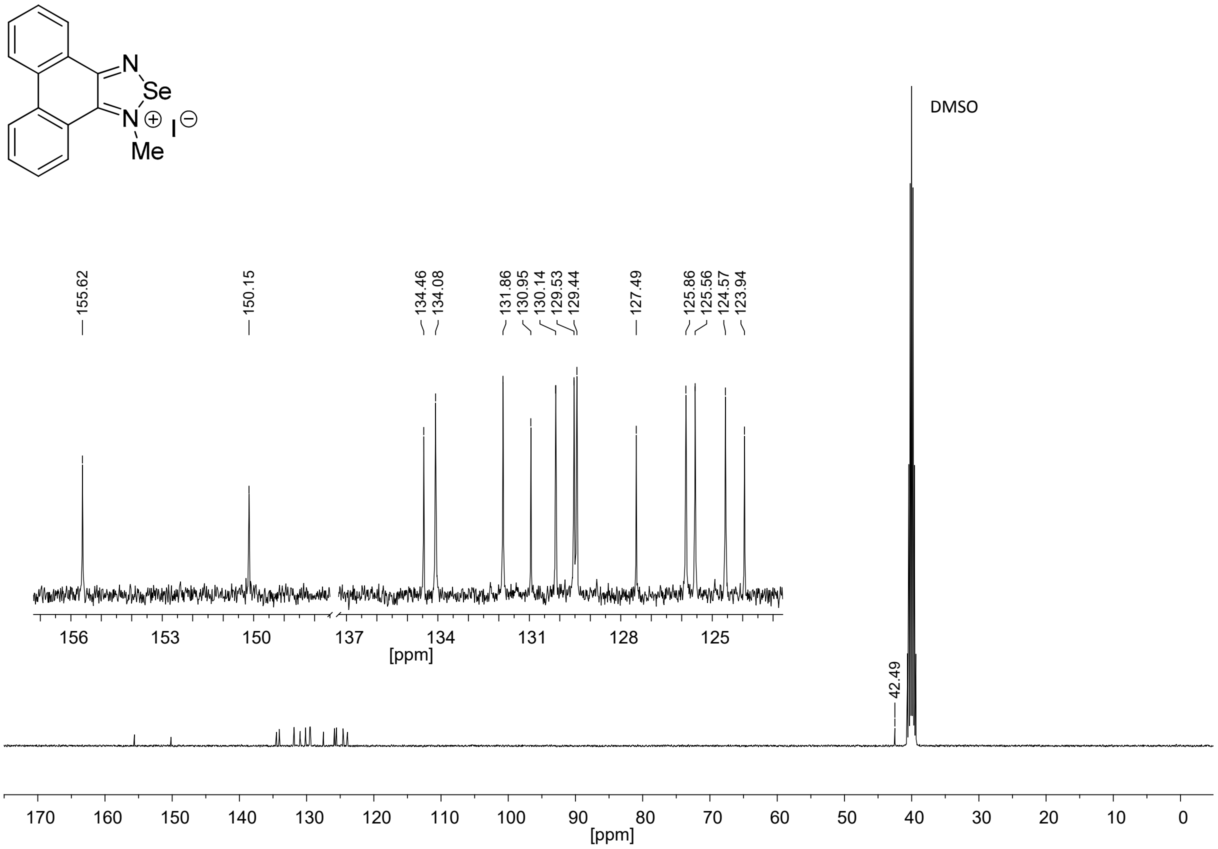

Figure S20. ^{13}C NMR spectrum of **4-MeI**.

Table S1. Selected crystallographic data.

	**1-MeI**	**2-MeTfO**	**2-MeI**	**4**	**4-MeTfO**	**4-MeI**
Chemical formula	$C_7H_7IN_2S$	$C_{16}H_{11}F_3N_2O_3S_2$	$C_{15}H_{11}IN_2S$	$C_{14}H_8N_2Se$	$C_{16}H_{11}F_3N_2O_3SSe$	$C_{15}H_{11}IN_2Se$
M, g·mol^{-1}	278.11	400.39	378.22	283.18	447.29	425.12
Crystal system	monoclinic	monoclinic	monoclinic	triclinic	monoclinic	triclinic
Space group	$P\ 2_1/c$	$I\ a$	$P\ 2_1/n$	P –1	$I\ a$	P –1
a, Å	7.5660(3)	6.7437(8)	6.3904(4)	3.9026(5)	6.7624(7)	9.8838(8)
b, Å	11.7008(6)	24.774(3)	11.1309(7)	14.651(3)	25.004(2)	11.1989(14)
c, Å	31.6418(14)	9.6689(12)	19.3308(13)	18.823(3)	9.6813(12)	13.6984(12)
α, °	90	90	90	94.065(13)	90	113.079(10)
β, °	93.677(4)	99.215(10)	92.724(5)	94.307(11)	99.918(9)	91.820(7)
γ, °	90	90	90	97.370(13)	90	95.733(8)
V, Å^3	2795.4(2)	1594.5(3)	1373.46(15)	1060.8(3)	1612.5(3)	1383.7(3)
Z	12	4	4	4	4	4
Temperature, K	293(2)	293(2)	293(2)	293(2)	293(2)	293(2)
Radiation type	Mo $K\alpha$	Mo $K\alpha$	Mo $K\alpha$	Mo $K\alpha$	Mo $K\alpha$	Mo $K\alpha$
ρ_{calc}, g·cm^{-3}	1.982	1.668	1.829	1.773	1.842	2.041
μ/mm^{-1}	3.600	0.388	2.470	3.513	2.511	4.931
$F(000)$	1584	816	736	560	888	808
Θ range/°	3.24–25.00	3.26–25.00	3.41–25.00	3.27–25.00	3.26–25.00	3.24–25.00
Completeness Θ/%	99.8	99.7	99.8	99.5	99.7	99.6
Reflections collected	21446	5425	8579	7102	6105	8916
Reflections unique	4909 [R_{int} = 0.0534]	2529 [R_{int} = 0.0412]	2420 [R_{int} = 0.0315]	3744 [R_{int} = 0.0820]	2551 [R_{int} = 0.0390]	4872 [R_{int} = 0.0592]
Data/restraints/ parameters	4909/0/301	2529/2/236	2420/0/175	4909/0/301	4909/0/301	4872/0/345
Goodness of fit on F^2	1.066	1.056	1.041	1.065	1.025	0.969
Final R_1 value ($I > 2\sigma(I)$)	0.0423	0.0523	0.0254	0.0800	0.0376	0.0553
Final wR_2 value ($I > 2\sigma(I)$)	0.0596	0.1159	0.0554	0.1625	0.0737	0.1096
Final R_1 value (all data)	0.0651	0.0762	0.0318	0.1340	0.0442	0.0981
Final wR_2 value (all data)	0.0653	0.1370	0.0585	0.1931	0.0764	0.1321
CCDC number	2034283	2034285	2034284	2034286	2034288	2034287

3. TD-DFT Calculations

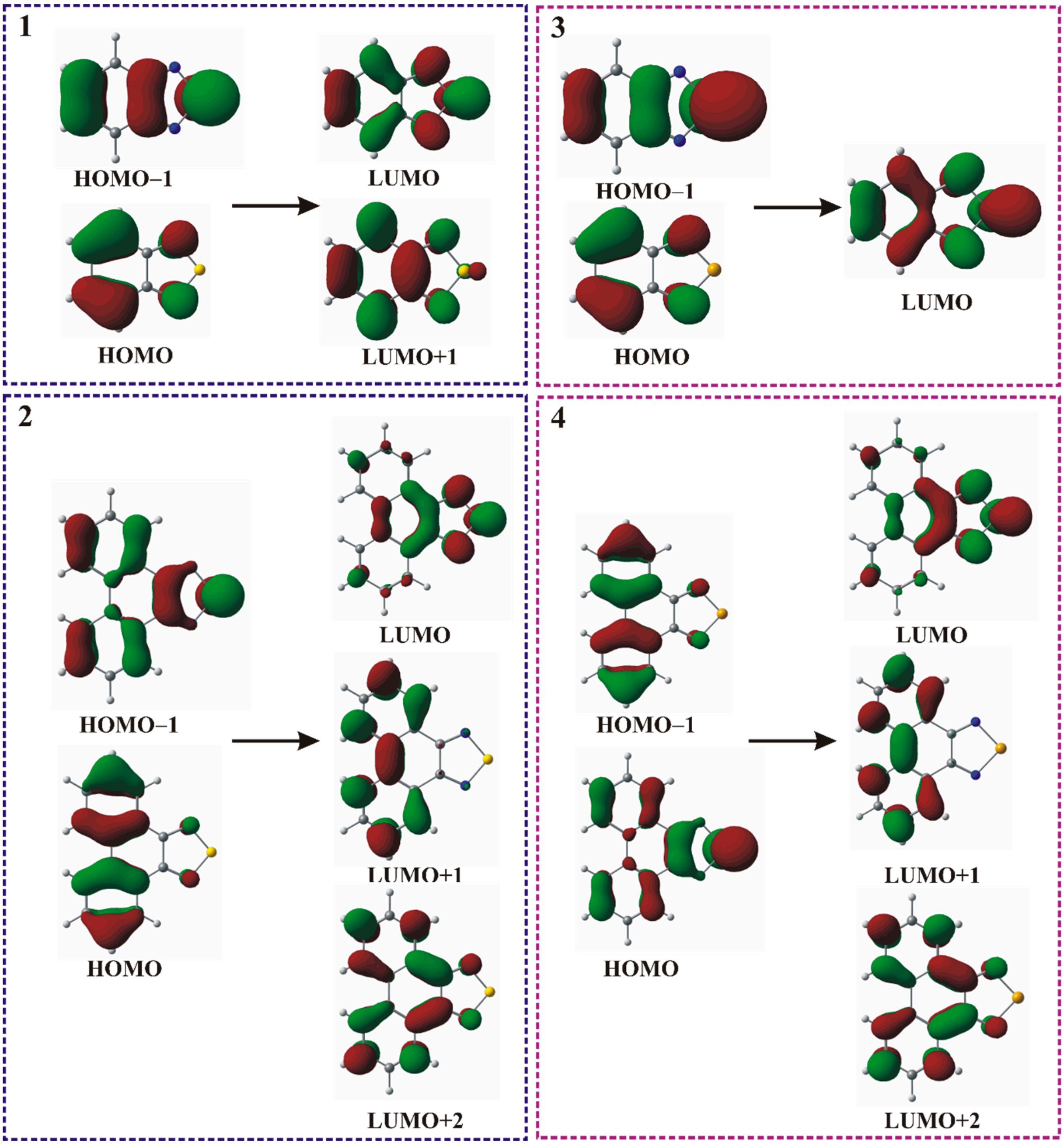

Figure S21. The most important molecular orbitals taking part in the electronic transitions for the neutral form of investigated molecules as calculated by the TD-DFT/B3LYP/6–31++G(d,p) methods (isosurface value equal to 0.04 a.u.$^{-3/2}$).

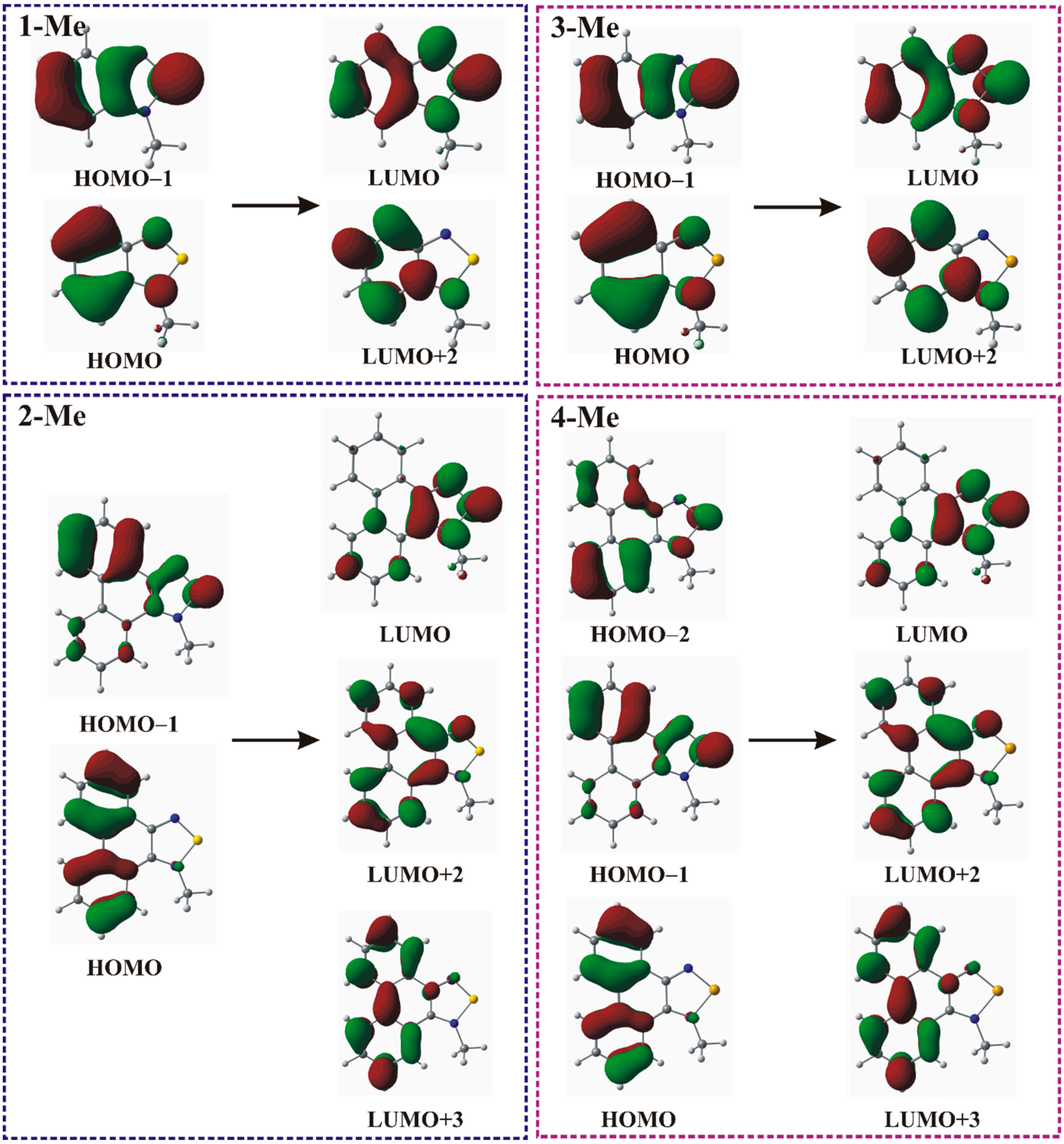

Figure S22. The most important molecular orbitals taking part in the electronic transitions for the cationic form of investigated molecules as calculated by the TD-DFT/B3LYP/6–31++G(d,p) methods (isosurface value equal to 0.04 a.u.$^{-3/2}$).

Table S2. The most important (oscillator strength > 0.03) electronic transitions of the neutral form of investigated compounds as calculated by the TD-DFT/B3LYP-D3/6–31++G(d,p) method in methanol.

Compound	Wavelength [nm]	Energy [eV]	Oscillator Strength	Main Contribution
		NEUTRAL FORM		
**1**	334.83	3.703	0.05	HOMO → LUMO
	281.83	4.399	0.29	HOMO–1 → LUMO<br>HOMO → LUMO+3
	213.80	5.799	0.05	HOMO–4 → LUMO<br>HOMO → LUMO+1
	205.53	6.032	0.39	HOMO–1 → LUMO+1<br>HOMO → LUMO+3
**2**	354.23	3.500	0.04	HOMO → LUMO
	330.03	3.757	0.26	HOMO–1 → LUMO
	284.75	4.354	0.03	HOMO–1 → LUMO+1
	272.46	4.441	0.07	HOMO–1 → LUMO+2<br>HOMO → LUMO+1
	252.63	4.908	0.83	HOMO–2 → LUMO+2<br>HOMO → LUMO+1
	252.31	4.914	0.20	HOMO–1→ LUMO+1<br>HOMO → LUMO+2
	239.14	5.184	0.26	HOMO–3 → LUMO<br>HOMO–3 → LUMO+1
	228.21	5.433	0.12	HOMO–2 → LUMO+2
	211.94	5.850	0.26	HOMO–2 → LUMO+1<br>HOMO → LUMO+5
	205.08	6.046	0.07	HOMO–3 → LUMO+1
**3**	356.33	3.479	0.04	HOMO → LUMO
	303.29	4.088	0.31	HOMO–1 → LUMO
	219.40	5.651	0.04	HOMO–4 → LUMO<br>HOMO → LUMO+2
	212.08	5.846	0.18	HOMO → LUMO+4
**4**	368.29	3.366	0.04	HOMO–1 → LUMO
	346.21	3.581	0.32	HOMO–1 → LUMO+2<br>HOMO → LUMO
	274.46	4.517	0.07	HOMO–1 → LUMO+1
	255.12	4.860	0.78	HOMO–1 → LUMO+1<br>HOMO → LUMO+2
	254.28	4.876	0.15	HOMO–3 → LUMO<br>HOMO–1 → LUMO+2
	250.82	4.943	0.21	HOMO–3 → LUMO<br>HOMO → LUMO+1
	233.07	5.320	0.05	HOMO–5 → LUMO<br>HOMO–1 → LUMO+2
	228.57	5.424	0.09	HOMO–2 → LUMO+2<br>HOMO → LUMO+5
	214.42	5.782	0.20	HOMO–1 → LUMO+5<br>HOMO–2 → LUMO+1
	206.25	6.011	0.14	HOMO–3 → LUMO+1<br>HOMO–1 → LUMO+8
	204.09	6.075	0.06	HOMO → LUMO+4<br>HOMO → LUMO+7
	200.60	6.181	0.04	HOMO–4 → LUMO+3

Table S3. The most important (oscillator strength > 0.03) electronic transitions of the cationic form of investigated compounds as calculated by the TD-DFT/B3LYP-D3/6–31++G(d,p) method in methanol.

Compound	Wavelength [nm]	Energy [eV]	Oscillator strength	Main contribution
		CATIONIC FORM		
[1-Me]$^+$	389.06	3.187	0.04	HOMO → LUMO
	295.07	4.202	0.31	HOMO–1 → LUMO<br>HOMO → LUMO+3
	219.61	5.646	0.08	HOMO–3 → LUMO<br>HOMO → LUMO+2
	207.30	5.981	0.33	HOMO → LUMO+3
[2-Me]$^+$	462.82	2.679	0.04	HOMO → LUMO
	390.21	3.177	0.18	HOMO–1 → LUMO
	337.01	3.679	0.13	HOMO–2 → LUMO<br>HOMO–1 → LUMO
	287.43	4.313	0.04	HOMO–3 → LUMO<br>HOMO–1 → LUMO+3
	285.40	4.344	0.04	HOMO–1 → LUMO+3<br>HOMO → LUMO+2
	270.14	4.590	0.09	HOMO → LUMO+3<br>HOMO → LUMO+2
	252.20	4.916	0.76	HOMO–1 → LUMO+2<br>HOMO → LUMO+3
	247.16	5.016	0.27	HOMO–1 → LUMO+3
	229.28	5.407	0.22	HOMO–2 → LUMO+2
	214.80	5.772	0.38	HOMO–7 → LUMO<br>HOMO–2 → LUMO+3
	211.99	5.849	0.09	HOMO–7 → LUMO<br>HOMO → LUMO+4
	208.29	5.952	0.05	HOMO–7 → LUMO<br>HOMO–1 → LUMO+4
[3-Me]$^+$	407.02	3.046	0.04	HOMO → LUMO
	314.33	3.944	0.34	HOMO–1 → LUMO<br>HOMO → LUMO+3
	227.99	5.438	0.06	HOMO–3 → LUMO
	216.54	5.726	0.09	HOMO–4 → LUMO
	207.10	5.987	0.06	HOMO–1 → LUMO+2<br>HOMO → LUMO+3<br>HOMO–4 → LUMO
	204.62	6.059	0.08	HOMO–2 → LUMO+1<br>HOMO–1 → LUMO+3
[4-Me]$^+$	475.10	2.610	0.03	HOMO → LUMO
	403.14	3.075	0.20	HOMO–1 → LUMO
	345.68	3.587	0.15	HOMO–2 → LUMO<br>HOMO–1 → LUMO
	291.90	4.247	0.04	HOMO–3 → LUMO
	284.34	4.360	0.04	HOMO → LUMO+2<br>HOMO → LUMO+3
	270.61	4.582	0.09	HOMO → LUMO+3<br>HOMO–1 → LUMO+2
	257.80	4.809	0.13	HOMO–4 → LUMO<br>HOMO–1 → LUMO+3
	252.72	4.906	0.70	HOMO–1 → LUMO+2
	246.27	5.034	0.15	HOMO–1 → LUMO+3<br>HOMO → LUMO+2
	229.69	5.398	0.18	HOMO–2 → LUMO+2
	221.94	5.586	0.12	HOMO–6 → LUMO<br>HOMO–5 → LUMO+1
	215.41	5.756	0.19	HOMO–5 → LUMO<br>HOMO → LUMO+4
	212.96	5.822	0.10	HOMO → LUMO+4<br>HOMO–6 → LUMO
	210.47	5.891	0.08	HOMO–1 → LUMO+4

Dipole moments (μ) and energies of frontier orbitals (E_{HOMO}, E_{LUMO}) were extracted directly from the data files following the geometry optimizations. Global reactivity descriptors are calculated by using the equations below [3]:

HOMO-LUMO gap energy:

$$\Delta E_{H-L\,gap} = E_{LUMO} - E_{HOMO} \quad (1)$$

Ionization potential:

$$IP = -E_{HOMO} \quad (2)$$

Electron affinity:

$$EA = -E_{LUMO} \quad (3)$$

Hardness:

$$\eta = \frac{E_{LUMO} - E_{HOMO}}{2} \quad (4)$$

Softness:

$$\zeta = \frac{1}{2\eta} \quad (5)$$

Electronegativity:

$$\chi = \frac{IP + EA}{2} \quad (6)$$

Electrophilicity index:

$$\psi = \frac{\chi^2}{2\eta} \quad (7)$$

4. X-ray Crystallography

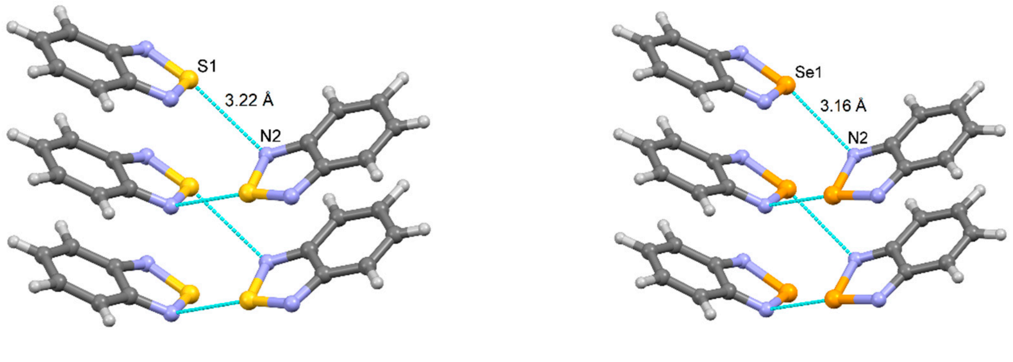

(a) (b)

Figure S23. Fragment of the catemer formed by molecules of **1** (**a**) and **3** (**b**).

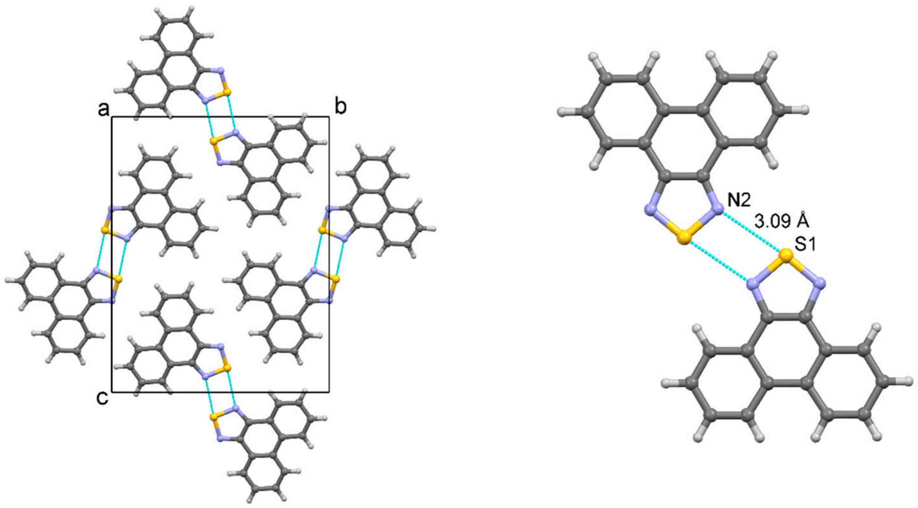

(a) (b)

Figure S24. (a) Crystal packing of **2** viewed along the [100] direction; **(b)** the **2**$_2$ dimer.

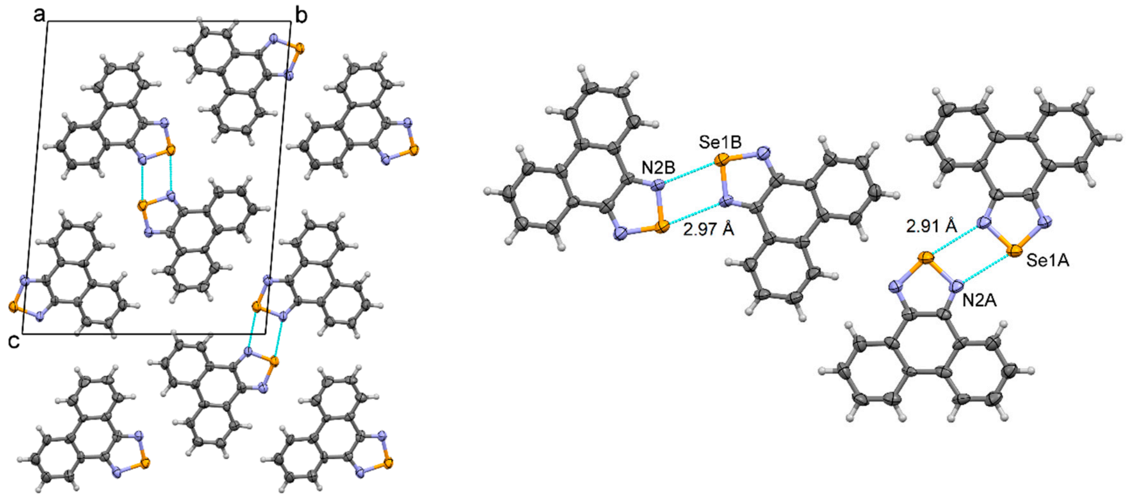

(a) (b)

Figure S25. (a) Crystal packing of **2** viewed along the [100] direction; **(b)** the two different **4**$_2$ dimers.

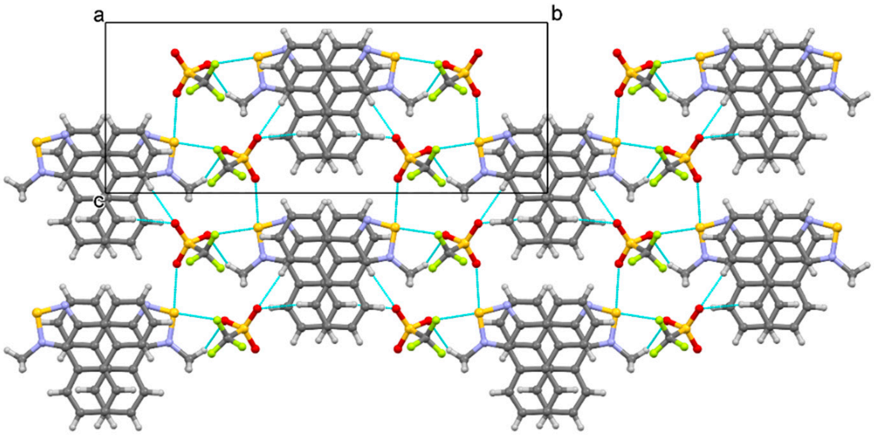

(a)

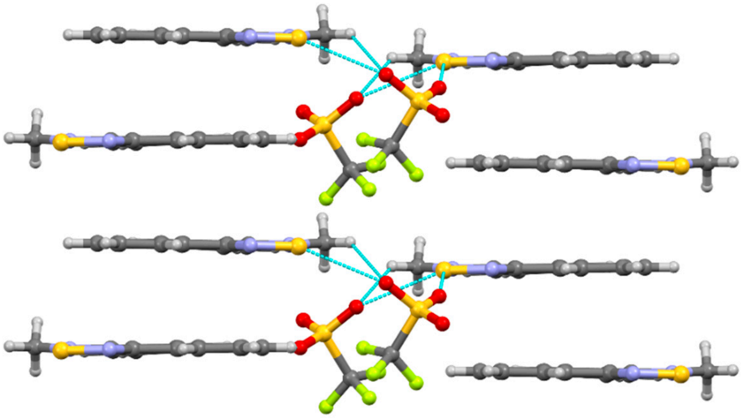

(b)

Figure S26. (a) Crystal packing of **2-MeTfO** viewed along the [100] direction; **(b)** the blocks of cations of **2-MeTfO** viewed along *c** axis.

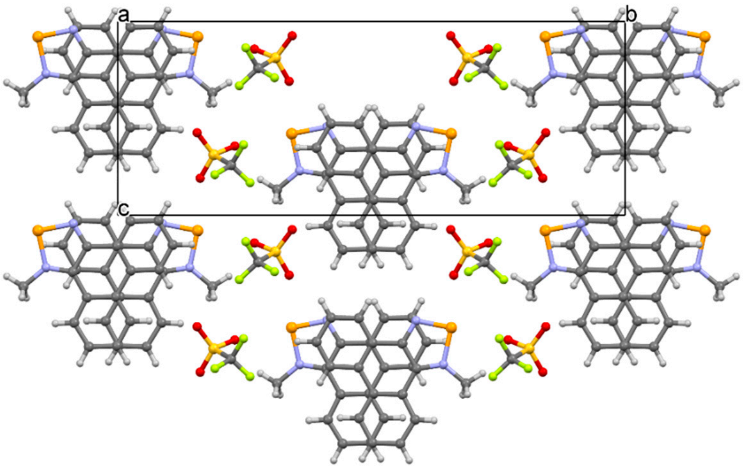

(a)

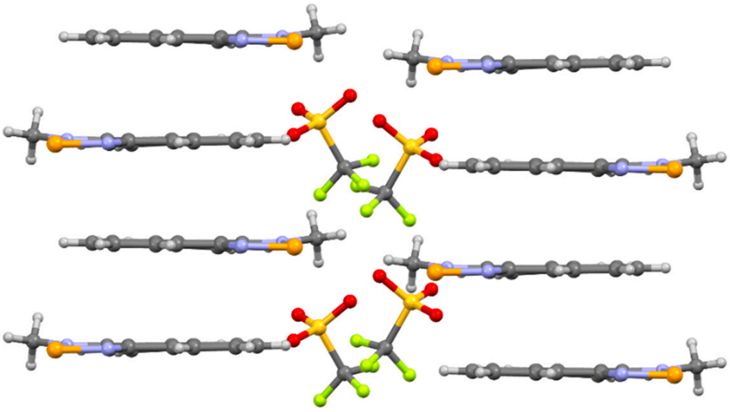

(b)

Figure S27. (a) Crystal packing of **4-MeTfO** viewed along the [100] direction; **(b)** the blocks of cations of **4-MeTfO** viewed along *c** axis.

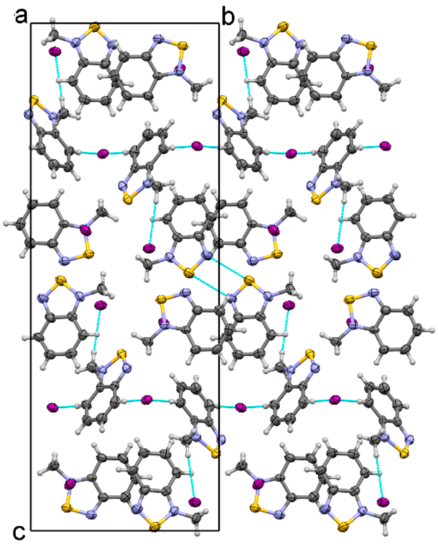

Figure S28. Crystal packing of **1-MeI** viewed along the *a* axis.

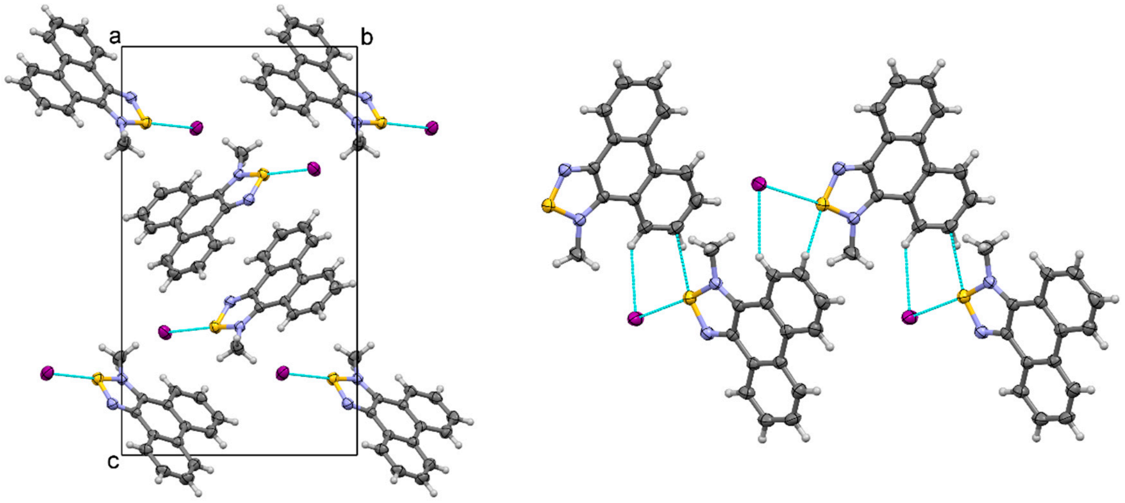

(a) (b)

Figure S29. (a) Crystal packing of **2-MeI** viewed along the *a* axis; **(b)** view of the polymeric chains.

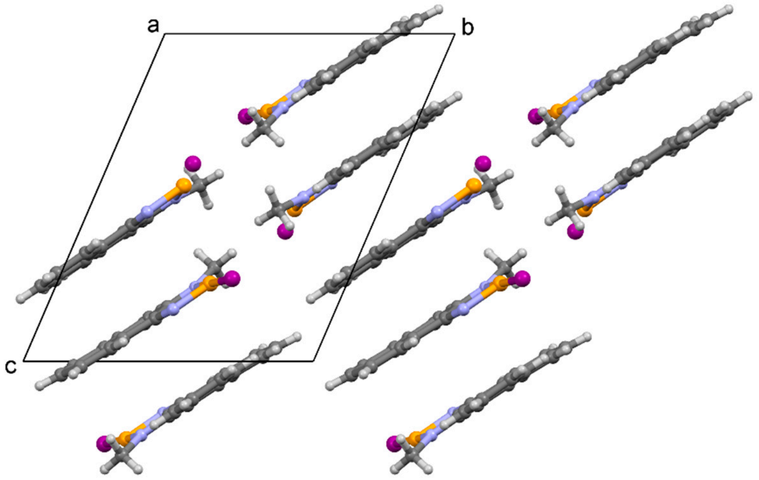

Figure S30. Crystal packing of **4-MeI** viewed along the *a* axis.

References

1. Risto, M., Reed, R. W., Robertson C. M., Oilunkaniemi R., Laitinen R. S., Oakley R. T., Self-association of the N-methyl benzotellurodiazolylium cation: Implications for the generation of super-heavy atom radicals *Chem. Commun.*, **2008**, *28*, 3278–3280.
2. Nunn A. J., Ralph J. T., 1254. Quaternisation of 2,1,3-benzothiadiazole and 2,1,3-benzoselenadiazole. Part I. Preparation of methyl- and ethyl-2,1,3-benzothiadiazolium and -benzoselenadiazolium salts, *J. Chem. Soc.*, **1965**, *1254*, 6769–6777.
3. Chattaraj P.K., Sarkar U., Roy D.R., Electrophilicity Index, *Chem. Rev.*, **2006**, *106*, 2065–2091.

© 2020 by the authors. Licensee MDPI, Basel, Switzerland. This article is an open access article distributed under the terms and conditions of the Creative Commons Attribution (CC BY) license (http://creativecommons.org/licenses/by/4.0/).
